# E-glass/kenaf fibre reinforced thermoset composites fiiled with MCC and immersion in a different fluid

**DOI:** 10.1038/s41598-022-24506-w

**Published:** 2022-11-25

**Authors:** Martinus Heru Palmiyanto, Eko Surojo, Dody Ariawan, Fitrian Imaduddin

**Affiliations:** 1 Mechanical Engineering Department, Faculty of Engineering, Universitas Sebelas Maret, Surakarta, 57126 Central Java Indonesia; 2 Mechanical Engineering Department, Sekolah Tinggi Teknologi Warga Surakarta, Sukoharjo, 57552 Central Java Indonesia

**Keywords:** Mechanical engineering, Composites

## Abstract

It is important to examine the long-term durability of glass-kenaf fibre reinforced phenolic resin composites when they are exposed to humid environments or submerged in water. Furthermore, the durability of such composites when immersed in different pH solutions have yet to be examined. As such, this present study examined the use of 4%, 8%, and 12% volume fractions (vfs) of microcrystalline cellulose (MCC) as a filler and reinforcement to improve the properties of glass fibre-kenaf reinforced phenolic resin composites. The flexural strength of these composites was examined both pre- and post-immersion in distilled water (pH 7), seawater (pH 8), and an acidic solution (pH 3) for 60 days. The diffusion mechanism, difussion coefficient, and water absorption concentration were also examined. The difussion coefficient and water absorption concentration occurred post-immersion in distilled water (pH7) and seawater (pH8) while the acidic solution (pH3) resulted in the highest loss of mass and size. Scanning electron microscopy (SEM) of the surfaces of the saturated composites indicated that fibre-matrix interfacial bonding was weak. However, composites that contained a higher vf of MCC exhibited stronger interfacial bonding between the matrix and constituents, thereby, reducing water absorption and diffusion. The flexural strength of the composite pre- and post-immersion was MCC12 > MCC8 > MCC4 > MCC0, in descending order of strength.

## Introduction

Green materials have attracted the attention of researchers and industries related to environmental and health issues. Natural fibre-reinforced composites are alternative structural materials that produce fewer emissions and environmental pollutants. However, the use of natural fibre composites in open areas is limited as they are hydrophilic and lack mechanical strength. Nevertheless, combining glass fibres and natural fibres have been found to reduce the likelihood of strain failure and increase interfacial adhesion strength^1,2^. It is critical to examine the long-term durability of natural fibre-reinforced polymer composites that are exposed to moist environments or submerged in water. This is because an ingress of water into the polymer causes material degradation; such as plasticisation, swelling, and glass transition; that reduce the transition temperature, flexural strength, and flexural modulus^3^. Furthermore, different types of water have different chemical properties, each of which affect the resistance of composite materials differently^4^. Composite materials may also be directly exposed to water in the form of rainwater, water, and seawater when in use.

Multiple studies have examined the mechanical properties of polymer composites that have aged in water, seawater, and acidic solutions^5–7^. Hydrogen bonds have been found to form between the water molecules and cellulose of natural fibre composites that are soaked in water. However, the penetration of water molecules into the fibre-matrix causes the composite to swell and decreases interfacial adhesion which, in turn, reduces tensile strength^8^. Interactions between the ionic mobility of various dissolved salts and the -OH groups of the polymer affect the penetration of seawater into a composite. When glass fibre reinforced plastic (GFRP) composites with an epoxy matrix are exposed to seawater, failures commonly occur in the ductile matrix and due to brittle fibres^6^. This is because the penetration of salt into the matrix causes it to swell, which results in tight contact between the matrix and the fibres. Prolonged soaking also causes alkaline oxides to leach, which degrades the E-glass fibres^6^. The acid concentration and diffusion behaviour of glass fibre-epoxy reinforced composites indicate that degradation occurred as the sulphuric acid concentration increased^9^. Furthermore, acidic environments have been found to damage microparticles on the surface of epoxy matrix composites. More specifically, acidic environments cause the bulk filler to flake off of the surface, which increases surface roughness and decreases the mechanical properties^10,11^.

Water absorption decreases the mechanical strength of composites via three mechanisms: (1) the diffusion of water molecules through defects in the surface of the matrix, (2) a capillary mechanism that flows water along the fibre-matrix interface which weakens the interfacial bond of the fibre-matrix, and (3) swelling the fibres which causes microcracks to form and propagate within the matrix^12^. The fracture energy increases when cracks encounter an impenetrable rigid barrier^13^. As such, nano- and micro-reinforcements; such as carbon nanotubes, ceramic particles, and glass particles; have been used as rigid fillers to increase the fracture energy^13,14^. However, although these fillers are biodegradable, they are expensive and hazardous to health.

Microcrystalline cellulose (MCC) is a rigid and environmentally-friendly natural material^16^. It is an attractive reinforcing filler for thermoset matrices as it is low density and possesses high specific strength and stiffness^17^. It is also a stronger and stiffer reinforcing filler as it has a larger surface area than natural cellulose, a large number of hydroxyl groups, and high crystallinity which increases the tensile, flexural, and impact properties of composites^18^. Microcrystalline cellulose (MCC) has a high degree of crystallinity as it is produced by reacting cellulose with an aqueous solution of strong mineral acid at boiling temperatures to remove the amorphous fraction and reduce the degree of cellulose chain polymerisation^19^. As such, it is resistant to water swelling and has good thermochemical stability^20^. Furthermore, MCC is a low density, non-toxic, biodegradable, and recyclable filler for polymer composites that contain other natural fibres^21^.

Although MCC is an efficient polymer matrix reinforcement filler, it is not without drawbacks; such as the durability of the mechanical properties of composites that are immersed in distilled water, seawater, and acidic solutions. Therefore, these obstacles must be overcome for these composites to perform well.

This present study examined the behaviour of E-glass-kenaf fibre reinforced thermoset composites reinforced with MCC and other fillers pre- and post-immersion in distilled water, seawater, and acidic solutions. The behaviour of the composites was investigated using water absorption concentration, diffusion mechanism, diffusion coefficient (*D*), and mechanical performance; specifically, flexural strength and flexural modulus. Scanning electron microscopy (SEM) was used to examine the effect of water absorption on the microstructure of the composites by comparing the surfaces of a control dry composite and that of immersed composites.

## Materials and methods

### Material

All the composites contained 25% volume fraction (vf) of phenolic resin-type thermoset binder and 12% vf of 5 mm E-glass and alkali-treated kenaf fibre reinforcement. Meanwhile, the filler consisted of 5% vf NBR, 5% vf molybdenum disulphide (MoS_2_), 10% vf graphite, 10% vf cashew dust, and 4% vf glass powder. Volume fractions (vf) of 0%, 4%, 8%, and 12% of Microcrystalline cellulose (MCC) with a particle size of 50 μm and a density of 0.26 g/cm^3^ were then added as a reinforcing filler to improve the mechanical properties of the composites while calcium carbonate (CaCO_3_) was added as a compensator. Variations in % volume of MCCand CaCO3 are shown in Table 1.

**Table 1:** Composition of the composites.

Symbol	Volume Fraction (%)		
	MCC	CaCO_3_	Basic mixture
MCC 0	0	29	71
MCC 4	4	25	71
MCC 8	8	21	71
MCC 12	12	17	71

The specifications of the ingredients were summarized in Table 2.

**Table 2:** List of raw materials and manufacturers.

Raw materials	Specifications	Manufacturer or supplier
Novolac phenolic resin	Powder size = 106 mm, melting point = 90 °C Density = 1,184 g/cm^3^	PT, Indopherin Jaya, Indonesia
Cashew dust	Powder size = 100 mesh, Density = 0.65 g/cm^3^	PT, Java Tohoku Industries, Indonesia
NBR	powder size = 120 μm, density = 1.04 g/cm^3^	LANXESS Corp
Graphite	Powder size = 100 mesh purity = 87%, density = 2,3 g/cm^3^	PT, Brataco Chemika, Indonesia
MoS_2_	Powder size = 100 mesh, purity = 98.5%, density = 2,3 g/cm^3^	PT, Brataco Chemika, Indonesia
CaCO_3_	Powder size = 100 mesh, density = 2.71 g/cm^3^	PT, Brataco Chemika, Indonesia
Microcrystalline cellulose (MCC)	Particle size = 50 μm, density = 0.26 g/cm^3^	Accent Microcell Pvt. Ltd
E-Glass fibers	WR 200, length = 5 mmDensity = 2.54 g/cm^3^	PT, Justus Kimiaraya, Indonesia
Kenaf fibers	Length = 5 mmdensity = 1.288 g/cm^3^	Alkaline-treated fiber in 4%wt of NaOH solution for 4 h
Glass powder	Powder size = 100 mesh Density = 2.47 g/cm^3^	The soda-lime glass waste was pounded with a ball mill to produce a size of 100 mesh, finally calcined at 500 °C for 3 h to remove impurities

**Figure 1:**
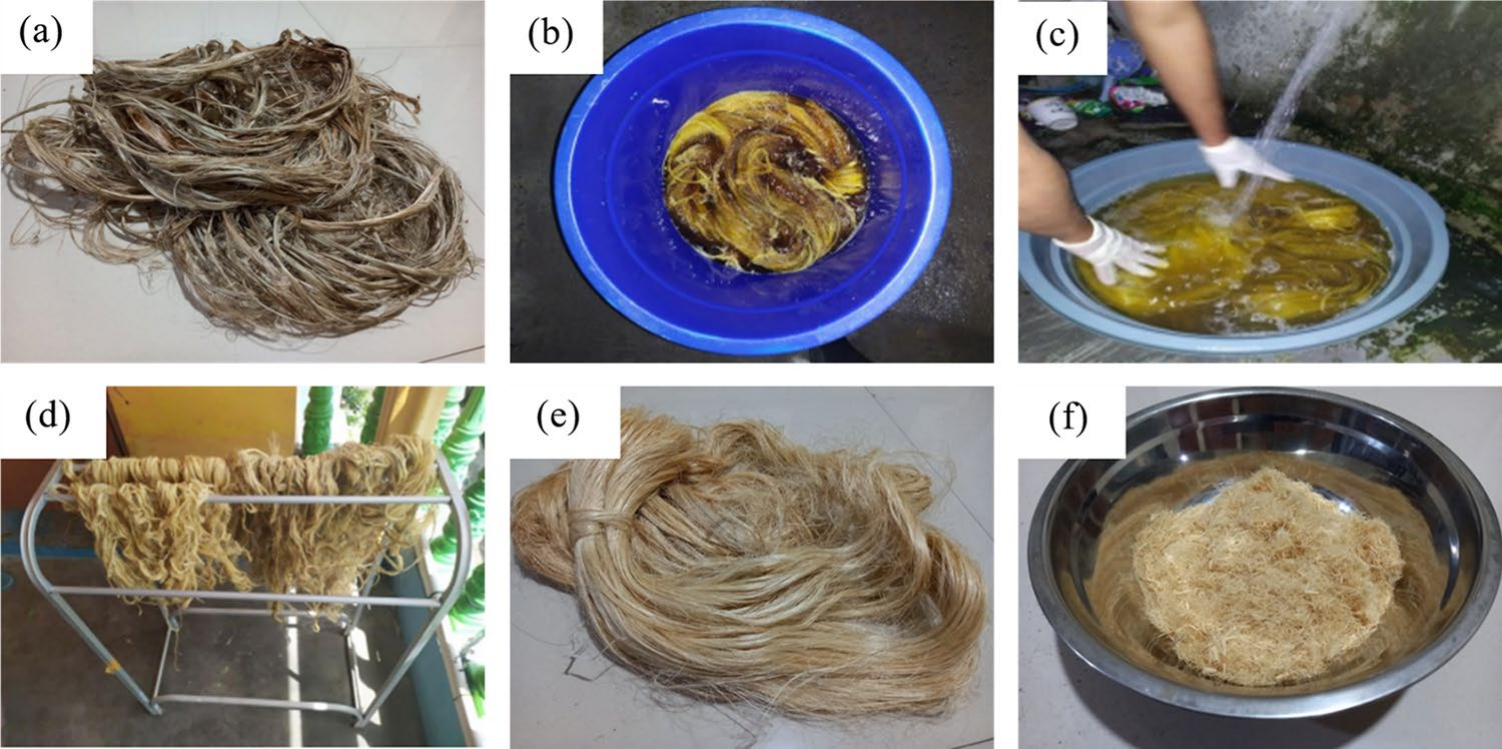
Kenaf fibre preparation process: (**a**) kenaf fibres post-extraction, (**b**) treatment in alkaline immersion, (**c**) pH neutralisation with an acetic acid solution and rinsing, (**d**) drying, and (**e**) cutting to short kenaf fibres.

The short kenaf fibres for composite reinforcement were prepared via the steps shown in Fig. 1. The kenaf fibres were extracted from the bark and core of kenaf stalks (Fig. 1a). The prepared kenaf fibres were then immersed in an alkaline solution 6% for 6 h. The kenaf fibres to aqua dest and sodium hydroxide (NaOH) crystal ratio was 1:5. Therefore, every 1 kg of kenaf fibres was immersed in an alkaline solution containing 4.7 L of aqua dest and 300 g of NaOH crystals (Fig. 1b). The kenaf fibres were then neutralised in 1% acetic acid to pH7 before they were rinsed with water (Fig. 1c). They were dried in indirect sunlight then heated in an oven at 60 °C for 8 h to remove excess moisture (Fig. 1d) before they were cut to measure 5 mm in length (Fig. 1e).

### Composite preparation

The composites were prepared according to predetermined vfs then stirred in two stages using a two-blade electric blender to produce a homogeneous mixture^22^. The powder form of a novolac phenolic resin; which is classified as a thermoset resin; was used to create the matrices. The composites were moulded according to the mould volume outlined in the ASTM D 790. As the materials had been prepared according to specific vfs, they were converted into weight fractions. These were weighed with the remaining constituent materials according to the predetermined composition variations. The filler and resin powders were first placed in a dry mixing tube then stirred for a total of 3 min at speed settings of 1, 3, and 5 for 1 min each. Add the fiberglass and kenaf fiber into the filler and resin mixture to continue mixing for a total of 5 min.

To prepare composites according to the ASTM D 790 for flexural testing, the necessary materials were placed in a mould then pressed at 20 MPa for 10 min. This was followed by hot pressing at 150 °C and 20 MPa for 10 min. During the first minute of the hot-pressing process, the pressure was released six times at 10 s intervals to discharge the gasses that composite materials produce during heating. The hot-pressing process was continued for the remaining 9 min. The composites were then post-cured to improve the cross-linking of their phenolic matrices^23^. This was accomplished by heating the composites from room temperature to 140 °C in an oven for 1 h. They were then heated from 140 °C to 180 °C for 6 h before they were allowed to cool from 180 °C to 30 °C for 30 min in the oven^24^.

### Density and void content

Equation (1) was used to calculate the density of the composites in vf according to the ASTM D792-98:

1 $$S_{p} = \frac{A}{{\left( {A + W - B} \right)}}{\text{gr}}$$

where $S_{p}$ is the actual mass of the composite, *A* = the mass of the composite without ballast in air, *B* = the mass of the composite and ballast wholly submerged in liquid, and *W* = the actual mass of the ballast wholly submerged in liquid. Equation (2) was used to calculate the actual composite density of the measurement results:

2 $$\rho_{a} = S_{p} * 0.9976{ } {\text{gr}}/{\text{cm}}^{{3}} { }$$

where $\rho_{a}$ is the actual density of the composites while the density of the distilled water was 0.9976 gr/cm^3^. Equation (3) was used to obtain the theoretical density of the composites in vf:

3 $$\rho_{t} = \rho_{1} .V_{1} + \rho_{2} .V_{2} \ldots . + \rho_{10} .V_{10} \;\;\;{\text{gr}}/{\text{cm}}^{{3}}$$

where *V* and ρ were the volume and density fractions, respectively, and the suffixes 1, 2, and 10 represented the constituents of the composites; such as fibre, matrix, MCC, and CaCO_3_.

The void content of the composites was determined according to the ASTM D-2734–70. Equation (4) was used to calculate the void volume fraction ($V_{v}$) of the composites:

4 $$V_{v} = \frac{{\rho_{t} - \rho_{a} }}{{\rho_{t} }}\%$$

where $V_{v}$ is the void volume fraction of the composites and $\rho_{t}$ and $\rho_{a}$ were the theoretical and actual densities of the composites, respectively.

### Water absorption

**Figure 2:**
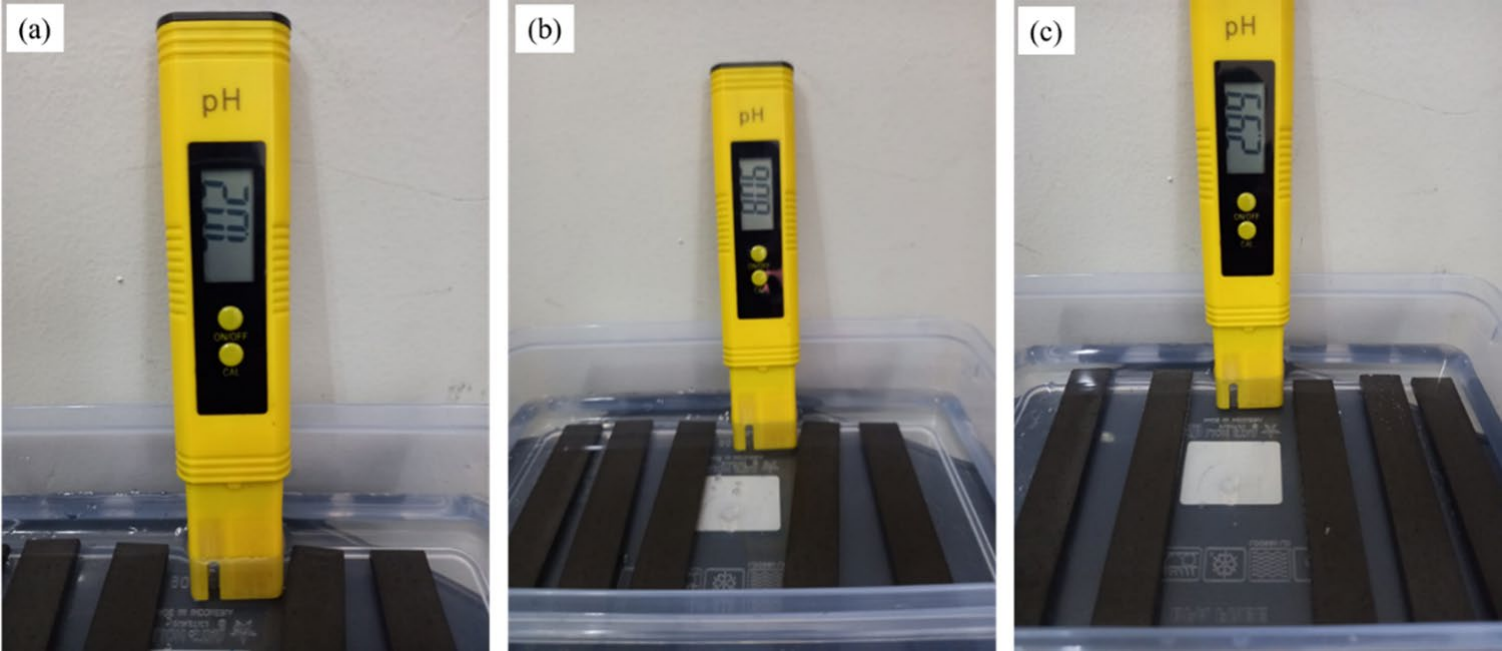
Three different immersion mediums: (**a**) distilled water (pH 7), (**b**) seawater (pH8), and (**c**) an acidic solution (pH 3).

The composites were immersed according to the ASTM D570 to determine the amount of water that the composites absorb^25^. Prior to immersion, the MCC composites were dried at 50 °C for 24 h then cooled in a desiccator until they were ready to be weighed and tested. The drying cycle was repeated until a mass constant of not less than 0.1 mg was obtained. A digital scale was used to measure the mass of the composites within an accuracy of 0.001 g. Three types of immersion mediums; distilled water (pH 7), seawater (pH 8), and an acidic solution (pH 3); were used (Fig. 2). The seawater medium was produced by mixing distilled water with artificial sea salt to obtain a pH of 8 while the acidic medium was produced by mixing distilled water with 99% acetic acid solution to obtain a pH of 3. The immersion mediums were inspected at 24-h intervals to ensure that they maintained constant pH levels. The composites were then soaked in the immersion mediums for 60 days at room temperature (23 ± 2 °C). The immersed composites were weight at 4-h intervals (240 ± 4 min) over the first 24-h period^26^. The interval between weight measurements was then increased to 24-h ± 1 h for the next 60 days.

Equation (5) was used to calculate the water absorption concentration (*M*_*m*_) from the changes in mass over time (*M*_*t*_) and the initial mass (*M*_*0*_)^12^:

5 $$M_{m} = \frac{{M_{t} - M_{0} }}{{M_{0} }} x 100\%$$

Equation (6) was used to calculate the percentage of change in size (*h*_*m*_), where (*h*_*t*_) was the final thickness and (*h*_*0*_) was the initial thickness.

6 $$h_{m} = \frac{{h_{t} - h_{0} }}{{h_{0} }} x 100\%$$

### Kinetics of the diffusion mechanism

**Figure 3:**
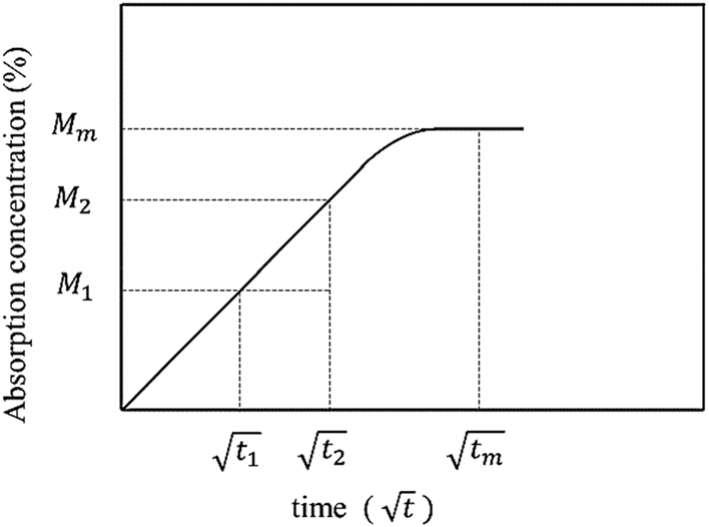
Water absorption curve according to Fick's laws of diffusion.

Figure 3 describes the diffusion properties of the composites according to Fick's laws of diffusion. Diffusion is the slope of the ratio between the water absorption concentration at a time divided by the saturated water absorption concentration by the square root of time.

The diffusion mechanisms were divided into three^29,30^: (1) typical behaviour according to Fick's law; when *n* = 0.5; and (2) when *n* = 1. The composites rapidly achieved water balance and maintained it as the immersion duration increased. The *n* was between 0.5 and 1 for anomalous diffusions. The water absorption diffusion mechanism was determined using the kinetic parameters of the slope (*n*) and intersection (*k*), which were obtained from the initial linear portion of the water absorption curve (log(*M*_*t*_/Max) vs. log (t)).

7 $$\log \left( {\frac{{M_{t} }}{{M_{{}} }}} \right) = \log \left( k \right) + n\log \left( t \right)$$

where *M*_*t*_ was the water absorption at the time (*t*) and *M*_*max*_ and *M*_*saturation*_ were the water absorption at the saturation point and the maximum moisture content, respectively.

Equations (8) and (9) were used to calculate the difussion coefficient (*D)* of absorbed water, where the water absorption of the first case, the *n* was close to 0.5, where *M*_*t*_/*M*_*max*_ was < 0.6 as calculated using Eq. (3). While the water absorption of the second case, the *n* was between 0.5 and 1, where *M*_*t*_/*M*_*max*_ was > 0.6 as calculated using Eq. (9) ^29^.

8 $$M_{t} = \frac{{4M_{max} }}{h}\sqrt {\frac{D.t}{\pi }}$$

9 $$M_{t} = M_{{}} .\left[ {1 - exp\left( { - 7.3\left( {\frac{D.t}{{h^{2} }}} \right)^{0.75} } \right)} \right]$$

where, *t* is the time and *h* is the thickness of the composite. The *D* can be calculated from the initial linear portion of the water absorption curve (i.e., the slope of *M*_*t*_ vs. *t*^*1/2*^. *h*^*-1*^)^30^.

### Flexural test

**Figure 4:**
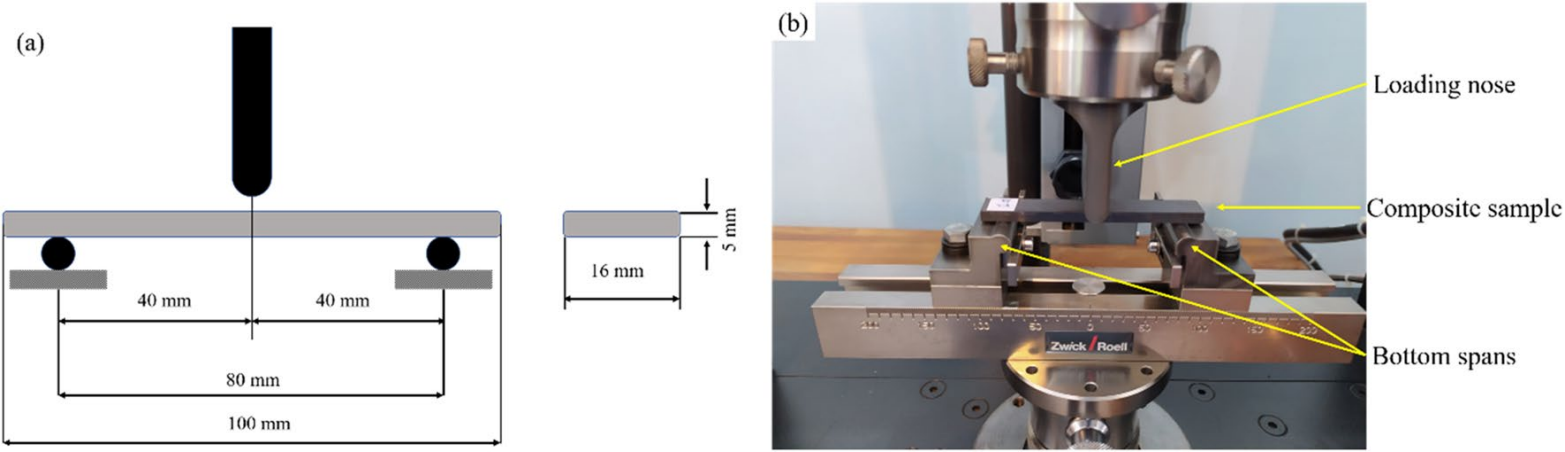
Flexural test according to the ASTM D790: (**a**) the size of the composite bar and the distance of the supports and loading nose and (**b**) the placement of the composite bar on the ZwickRoell® Z020 universal testing machine.

As per the ASTM D 790, the flexural strength of the composites was measured using the three-point bending method. The dimensions of the flexural test composite bars were 100 mm × 16 mm × 5 mm. Figure 4a depicts the support distance and loading nose adjusted according to the ASTM D790.

The flexural strength test was conducted using a Zwick/Roell Z020 universal testing machine (Fig. 4b). The rectangular cross-section of the composite bars was rested on two supports and loaded via the loading nose halfway between the supports until the composite bar collapsed. The experiment was repeated five times for each composite bar to obtain more accurate results.

The flexural tests were conducted at a span length of 80 mm and a crosshead speed of 5 mm/minute. The tested samples included a control sample and a dried immersion sample. The control sample served to provide a comparison of the flexural strength of the dried immersion sample. Prior to flexural testing, the immersed samples were dried in an oven at 60 °C for 24 h. Each flexural test was repeated five times and the average value was recorded.

## Results and discussion

### Physical characterisation of microcrystalline cellulose (MCC)

**Figure 5:**
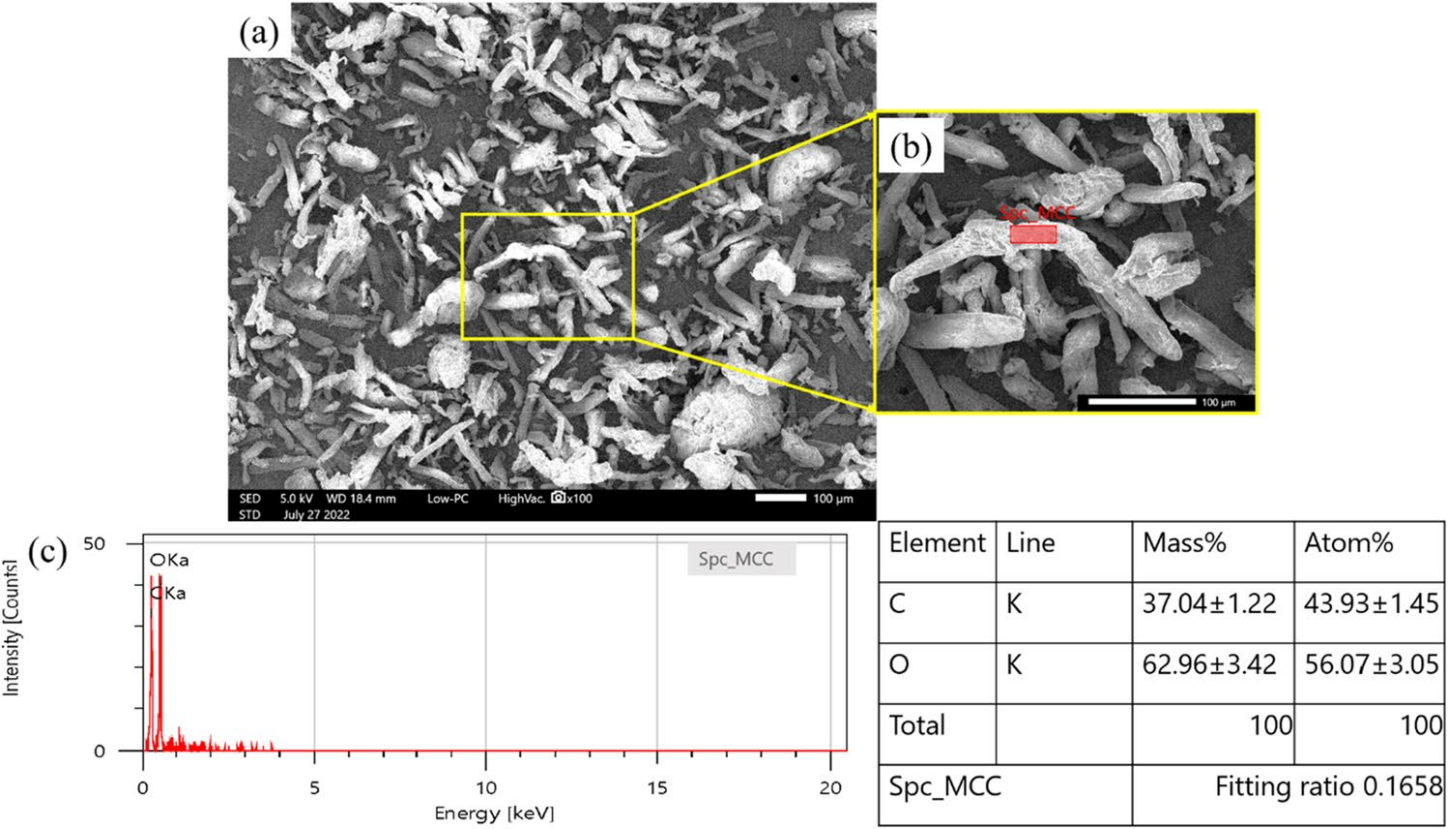
SEM–EDX analysis of the MCC elements: (**a**) the SEM micrograph of the shape surface morphology of the MCC powder, (**b**) the EDX point area, and (**c**) the EDX spectrum and right table for the atomic percentages and weights of the various MCC elements.

Scanning electron microscopy (SEM) with energy dispersive X-ray (EDX) analysis was used to characterise the chemical composition, shape, concentration, and morphology of the MCC (Fig. 5).

As seen in Fig. 5a, the SEM micrograph indicates that the MCC had the shape and surface morphology of MCC, which resembles needle-like structures of different lengths and widths. As seen in Fig. 5b, the MCC size analysis indicates that the MCC was 71.08 to 184.02 m in length and 11.89 to 41.33 m in width. Figure 5a also shows the distribution of MCC fibres and some agglomerated MCC fibres.

The area seen in Fig. 5b was analysed using EDX. Figure 5c provides the EDX spectrum while the table on the right shows the atomic percentages and weights of the various elements. The EDX spectrum of the analysed region showed that the MCC contained 37.04% of carbon (C) and 62.9% of oxygen (O).

### Density and void content

Table 3 depicts the density of each composite complete with sample codes. As seen, the MCC12 composite had the lowest density while the MCC0 composite had the highest. Therefore, higher MCC vfs decrease composite density theoretically and practically. The composites were highly dense due to the high CaCO_3_ vf, where the CaCO_3_, which was used as a compensator, was denser than the MCC.

**Table 3:** Density and void content of the composites.

Specimen code	Measured density $(gram/cm^{3} )$	Theoretical density $(gram/cm^{3} )$	Volume fraction of void $(\%$)
MCC 0	1.826 ± 0.012	1.966	6.855 ± 0.618
MCC 4	1.806 ± 0.005	1.869	3.116 ± 0.666
MCC 8	1.748 ± 0.017	1.772	1.085 ± 0.646
MCC 12	1.668 ± 0.002	1.675	0.126 ± 0.097

Voids are closed pores that form in the composites when air becomes trapped at the beginning of the manufacturing process^31^. Thermoset composite matrices are made by mixing resin powder and other constituents in a mould. The constituents are wetted by heating the resin and the constituents in the mould. Poor wetting inhibits matrix percolation and the movement of trapped air during the reaction. Voids are primarily formed by the inhomogeneous distribution of constituents resulting in non-uniform permeability^32^. The presence of volatile components and impurities may also cause voids to form during the curing process. As seen in Table 3, the addition of 4%, 8%, and 12% vf of MCC decreased the $V_{v}$. The MCC12 sample had a void content of 0.19% vf. The decrease in the current volume of voids with particle loading can be attributed to the ability of the MCC to distribute and disperse in the matrix. As the matrix partially covers the MCC particles, it alludes to the extraordinary possibility of interfacial interaction between the surfaces of the matrix and the MCC particles^12,17^.

### Water absorption analysis

**Figure 6:**
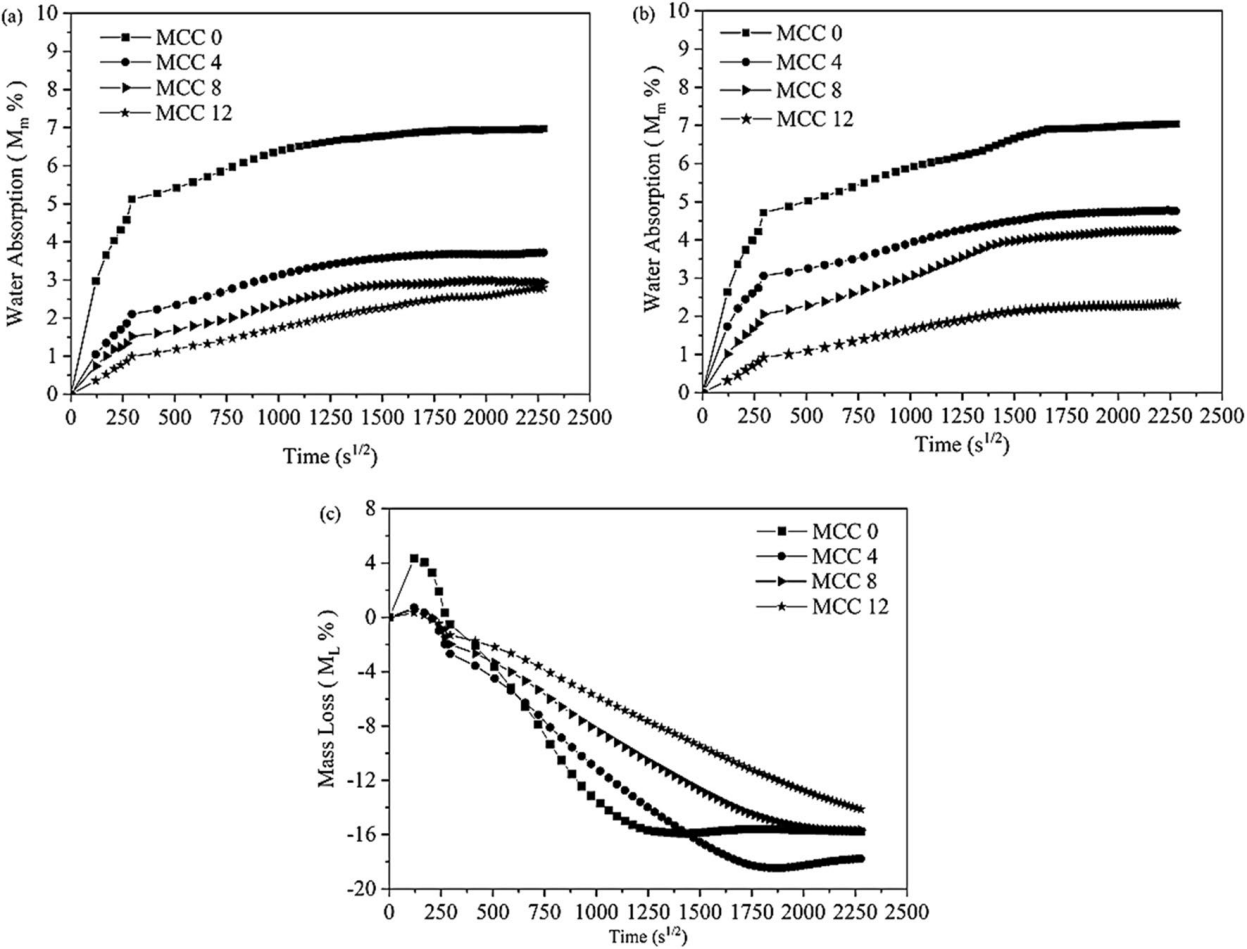
The curve of water absorption (*Mm*) composites immersed in: (**a**) distilled water, (**b**) seawater, and (**c**) an acidic solution.

Figure 6 shows the behaviours of the various MCC composites (0%, 4%, 8%, and 12%) that had been immersed in distilled water (pH7), seawater (pH8), and an acidic solution (pH3) for 60 days.

The weight percentage (*M*_*m*_) of composites immersed in distilled water and seawater increased, indicating that the composites absorbed water (Figs. 6a and b). The initial water absorption mass increased linearly with the square of root time then increased slowly until the end of the observation period. Meanwhile, the *M*_*m*_ of the composite immersed in the acidic solution decreased as the immersion duration increased. Figure 6c depicts the percentage of mass loss (*M*_*L*_) and time root (s^1/2^) curves.

Figures 6a and b depict the curves of the percentage of water absorbed (*M*_*Max*_) by the MCC composites. As seen, composites with 4%, 8%, and 12% vf of filler-reinforcements (MCC4, MCC8, and MCC12) absorbed less water than to the composite with no filler-reinforcement (MCC0). Table 4 provides the *M*_*Max*_ at the initial immersion period (t_7_ = 24 h) and at the end of the immersion period (t_end_ = 60 days)**.**

**Table 4:** Percentage of water absorption and mass loss of composite.

Spesimen code	Percentage mass absorption					Percentage mass loss		
	t = 24 h (Mt, %)		t = 60 day (M_max,_%)			t = 24 h (Mt, %)		t = 60 day (M_max,_%)
	Distilled water	Seawater	Distilled water		Seawater	Acidic solution		
MCC 0	5.11 ± 0.87	4.71 ± 0.15	6.95 ± 0.70	6.95 ± 0.27	− 6.43	± 0.74	− 15.75	± 1.45
MCC 4	2.10 ± 0.75	3.05 ± 1.60	3.71 ± 0.75	4.77 ± 1.95	− 3.40	± 0.46	− 17.80	± 0.65
MCC 8	1.52 ± 0.74	2.05 ± 1.30	2.93 ± 0.99	4.25 ± 1.49	− 2.58	± 0.45	− 15.69	± 0.53
MCC 12	0.99 ± 0.09	0.91 ± 0.15	2.77 ± 0.24	2.32 ± 0.33	− 1.64	± 0.27	− 13.87	± 1.33

The MCC0 composite had an *M*_*Max*_ of 6.953% at t_end_ = 60 days in distilled water and seawater while the MCC12 composite had the lowest *M*_*Max*_. Furthermore, the *M*_*Max*_ of the MCC12 was 80% lower than that of the MCC 0 composite at t_7_ = 24 h. This difference decreased to 60% at t_end_ = 60 days in distilled water and seawater.

As seen in Fig. 6c, the *M*_*Max*_ of MCC composites immersed in the acidic solution increased rapidly at t_1_ = 4 h. Furthermore, the *M*_*L*_ of the composites increased as the duration of immersion in the acidic solution increased. Table 4 provides the *M*_*L*_ of the composites post-immersion in the pH 3 acidic solution. As the *M*_*L*_ was calculated at t_1_ = 4 h, *M*_*t1*_ is the initial mass. By comparing the *M*_*L*_ of the MCC0 and MCC12 composites, it is evident that the addition of 12% vf of MCC decreased the *M*_*L*_ at t_7_ = 24 h by 60% and at t_end_ = 60 days by 40%.

**Figure 7:**
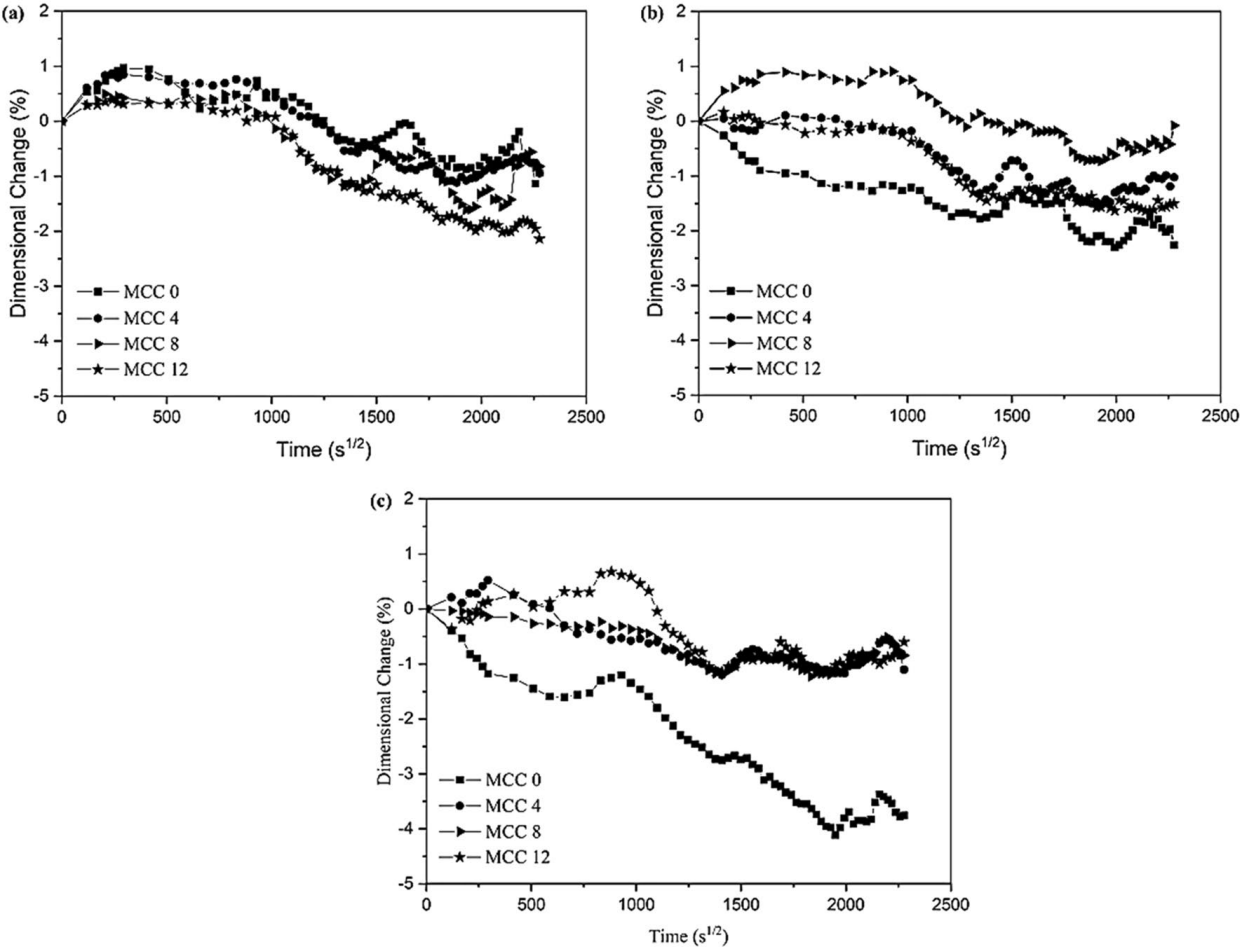
The curve of dimensional changes (*h*_*m*_) of composites immersed in (**a**) distilled water (pH 7), (**b**) seawater (pH 8), and (**c**) the acidic solution (pH 3).

As seen in Fig. 7, the curve of the percentage of change in mass at t_end_ = 60 days in is not entirely balanced. The composites achieved moisture content equilibrium only when they gained less than 0.01% in weight daily. Under equilibrium conditions, the absorbed water is known as free water and does not cause any dimensional changes (*h*_*m*_)^33^. Figure 7 depicts the correlation between the *h*_*m*_ and duration of immersion in (a) distilled water (pH 7), (b) seawater (pH 8), and (c) the acidic solution (pH 3).

The composites were measured at three different sites and recorded in millimetres. Equation (3) was then used to calculate the *h*_*m*_. Table 5 presents the *h*_*m*_ of the composites immersed in the three different mediums.

**Table 5:** The dimensions changes of composite (h_m_) in the immersions distilled water, seawater, and acid water.

Spesimen code	Dimensional change (hm, %)											
	t = 24 h						t = 60 day					
	Distilled water		Seawater		Acidic solution		Distilled water		Seawater		Acidic solution	
MCC 0	0.97	± 0.30	− 0.90	± 1.08	− 1.18	± 0.82	− 0.75	± 0.79	− 2.02	± 0.92	− 3.70	± 1.06
MCC 4	0.85	± 0.69	− 0.07	± 0.85	0.52	± 0.92	− 0.77	± 0.96	− 0.99	± 1.09	− 0.68	± 0.96
MCC 8	0.42	± 0.61	0.86	± 0.87	− 0.14	± 0.62	− 0.56	± 0.98	− 0.35	± 0.94	− 0.73	± 0.95
MCC 12	0.31	± 0.80	− 0.03	± 0.89	0.14	± 0.63	− 1.87	± 1.05	− 1.56	± 0.82	− 0.83	± 0.82

A positive *h*_*m*_ indicates an increase in specimen volume while a negative *h*_*m*_ indicates a decrease. Composites immersed in distilled water (pH 7) experienced the highest positive *h*_*m*_ (0.97%) at t_7_ = 24 h. However, the *h*_*m*_ decreased as it approached t_end_ = 60 days, indicating that the specimen volume decreased. The MCC0 composite exhibited the highest negative *h*_*m*_ (3.70%) in the acidic solution (pH3). As seen in Fig. 7 and Table 5, the *h*_*m*_ of all the composites immersed in distilled water (pH7), seawater (pH8), and acid solution (pH3) decreased at t_end_ = 60 days. This indicates that composites experienced physical changes that may be attributed to reactions with the immersion fluids^34^. According to Iordanskii^35^, loss of molecular weight is the most important parameter with which to monitor degradation. This is in addition to *h*_*m*_ and loss of mechanical strength. As such, these physical changes indicate that the constituent materials of the composites degraded during immersion.

A comparison Figs. 7 and 6 indicate that the *h*_*m*_ and the percentage of change in mass exhibited similar trends. The MCC0 composite immersed in distilled water had the highest *h*_*m*_ and *M*_*m*_ while the MCC0 composite immersed in the acidic solution had the lowest *h*_*m*_ and *M*_*m*_. A decrease in the percentage of weight indicates a loss of mass. Under the same conditions, water absorption into the composite results in a change in dimensions. Therefore, *M*_*m*_ affects the *h*_*m*_ of MCC composites.

The addition of 4%, 8%, and 12% vf of MCC improved the *h*_*m*_ and *M*_*m*_ of the composites immersed in distilled water, seawater, and the acidic solution better than the MCC0 composite. As seen in Tables 4 and 5, the addition of MCC as a filler for composite reinforcement increased the resistance of composites immersed in distilled water, seawater, and the acidic solution. This phenomenon may occur due to the natural properties of MCC; such as good permeability, compatibility, and compressibility; all of which are required to manufacture composites^17,36^. Furthermore, the use of MCC as filler-reinforcement is believed to increase the interfacial bond between the matrix and the constituents, thereby limiting the *h*_*m*_ and *M*_*m*_ of composites^37,38^.

Although MCC increased the stability of the mass of the composites (Table 4), the *h*_*m*_ of the MCC12 composite at t_end_ = 60 days (Table 5) was unsatisfactory. More specifically, the *h*_*m*_ of the MCC12 composite immersed in distilled water, seawater, and the acidic solution was higher than that of the MCC4 and MCC8 composites. The higher the molecular weight of MCC, the higher the potential to bind more water molecules which, in turn, increased the degradation of the composite. Therefore, the higher *h*_*m*_ of the MCC12 composite could be attributed to the higher molecular weight of MCC that it contained.

Composites degrade as its polymers react with the surrounding environment and the molecules naturally or artificially break into smaller molecules^39^. Polymers generally degrade very slowly when exposed to oxygen via an autocatalytic reaction. However, the presence of several substances that catalyse the oxidation process can accelerate the reaction^35^. Studies on the effect of pH on polymer degradation have concluded that neutral pH solutions have the highest polymer chain-breaking strength while low and high pH solutions cause rapid degradation to occur^39,40^.

This present study found that composites made of a phenolic resin matrix and its constituents decomposed more rapidly when immersed in an acidic solution (pH 3) than in distilled water (pH 7) and seawater (pH 8). A higher MCC content is also believed to *M*_*m*_, which accelerates the decomposition of the matrix via oxidation.

### Kinetic analysis of water sorption

**Figure 8:**
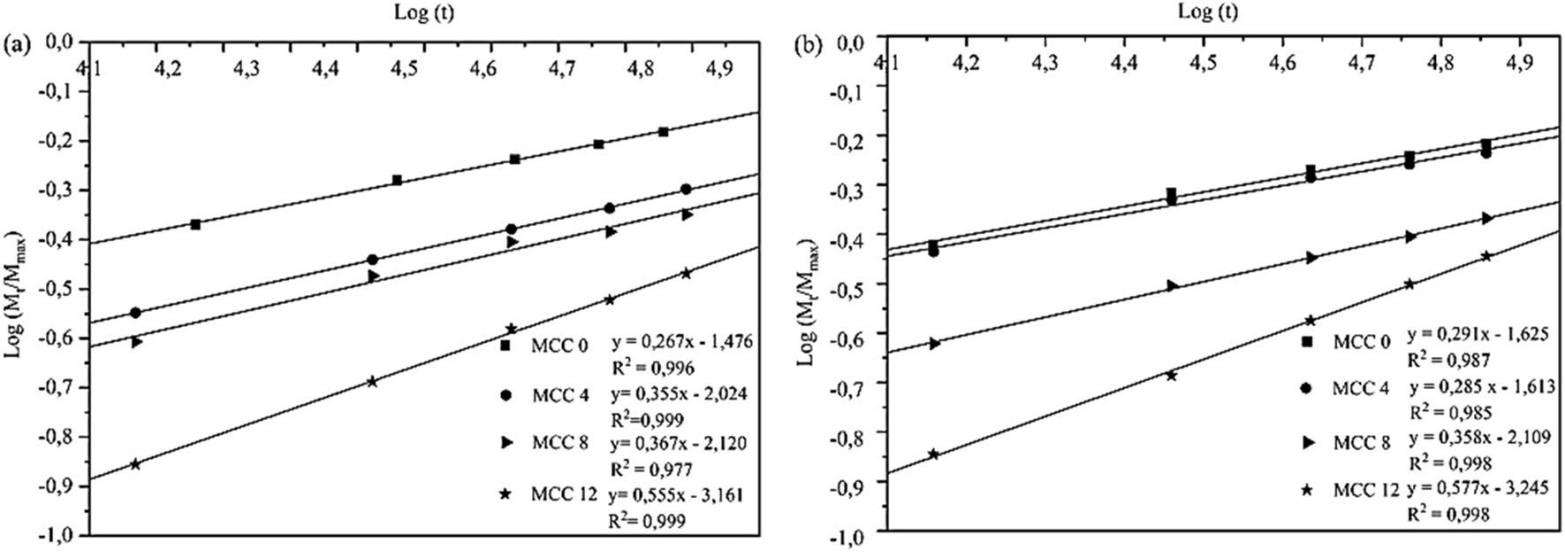
The curve of dimensional changes (*h*_*m*_) of composites immersed in (**a**) distilled water (pH 7), (**b**) seawater (pH 8), and (**c**) the acidic solution (pH 3).

Figure 8 presents the diffusion mechanism of the slopes as well as the curve of log (M_t_/M_max_) versus log (t) of MCC composites immersed in distilled water and seawater.

As seen in Figs. 8a and b, the trend of the water absorption mechanism curve can be considered a Fickian diffusion process^41^. The diffusion mechanism was divided into three cases^42^: Case I, where the slope (*n*) = 0.5 causing the rate of water molecule absorption to be slower than the mobility of the polymer relaxation process; Case II, where the *n* = 1.0 causing the rate of water molecule absorption to be faster than the polymer relaxation process; and Case III where the composites quickly achieve and maintain moisture content equilibrium throughout the immersion period. Non-Fickian diffusion (anomaly) occurs when 0:5 < *n* < 1:0. Transitions, between Fickian and non-Fickian diffusion, were observed to determine the proportion of the diffusion rate of the water molecules and the relaxation of the chains of the polymer molecules^43^.

The *n* and the intercept (*k*) were determined using the graphs seen in Figs. 8a and b and calculated using Eqs. (2) and (3). Table 5 presents the absorption constants of distilled water and seawater for all MCC composites. The *n* and *k* of distilled water (pH7) and seawater (pH8) were almost identical. This could be due to the closeness in pH of both mediums. As seen in Table 6, the diffusion constants (*n*) of the MCC0, MCC4, and MCC8 composites were significantly < 0.5, which indicates a deviation from Fickian diffusion^42^. This deviation could be due to fibre swelling, weakening of the interfacial bonds of the fibre matrix, microcracking, and leaching because of the different water absorption mechanisms within the kenaf fibre reinforced composites. Meanwhile the *n* of the MCC12 composite was n > 0.5. However, as the n was close to 0.5, the characteristics of the diffusion mechanism could be considered Fickian.

**Table 6:** Water absorption parameter and difussion coefficient values for all MCC composite.

Spesimen code	Fluid Immersion	Maximum moisture Mmax (%)	Difussion coefficient (m.s^(−1/2)^)	n (slope)	k (intercept)	R^2^
MCC 0	Distilled water	6.953	4.648E-13	0.267	1.475	0.996
MCC 4	Distilled water	3.710	4.204E-13	0.355	2.023	0.999
MCC 8	Distilled water	2.938	3.565E-13	0.367	2.12	0.997
MCC 12	Distilled water	2.770	3.315E-13	0.554	3.16	0.999
MCC 0	Seawater	6.953	4.493E-13	0.291	1.624	0.987
MCC 4	Seawater	4.780	3.879E-13	0.285	1.612	0.985
MCC 8	Seawater	4.253	3.094E-13	0.358	2.109	0.998
MCC 12	Seawater	2.322	3.868E-13	0.576	3.247	0.998

The *D* in Fick's model represents the ability of water molecules to penetrate a composite. Equation (8) was used to estimate the *D* shown in Table 6. The higher the *D*, the higher the maximum water absorption. The *D* of distilled water (pH7) and seawater (pH8) of all the MCC composites were similar. This could be due to the closeness in pH of both mediums. The *D* was found to decrease as the MCC vf of the composites increased (0, 4, 8, and 12). The MCC0 composite had the highest *D* while the MCC12 composite had the lowest.

Many factors; such as fraction volume fibres, free volume, solution concentration, concentration gradient diffusion, hydrolysis reactions, plasticisation, defects, and voids on the surface affect the absorption and diffusion of water molecules in thermoset resin-based composites^28,44,45^. The rapid absorption of water over a relatively short time is due to the hydrolysis of the free water molecules in the solution and the functional groups on the surface of the resin^46^. The water molecules are rapidly adsorbed by the pores and holes on the surface of the resin under environmental pressure and concentration gradient. As seen in Table 3, the MCC0 composite had the highest void content and the void content decreased as the MCC vf increased. The *D* also decreased as the MCC vf increased. This correlates with the void volume, which is affected by the compatibility and compressibility of the MCC particles which provide size and mass stability. Water may be transported into polymer composite via interfacial gaps between the fibre and the matrix as well as air voids in the matrix^45^. The addition of MCC as a filler-reinforcement is believed to increase the interfacial bond between the matrix and the constituents of the composite, thereby limiting water absorption and diffusion rate.

### Surface morphology

**Figure 9:**
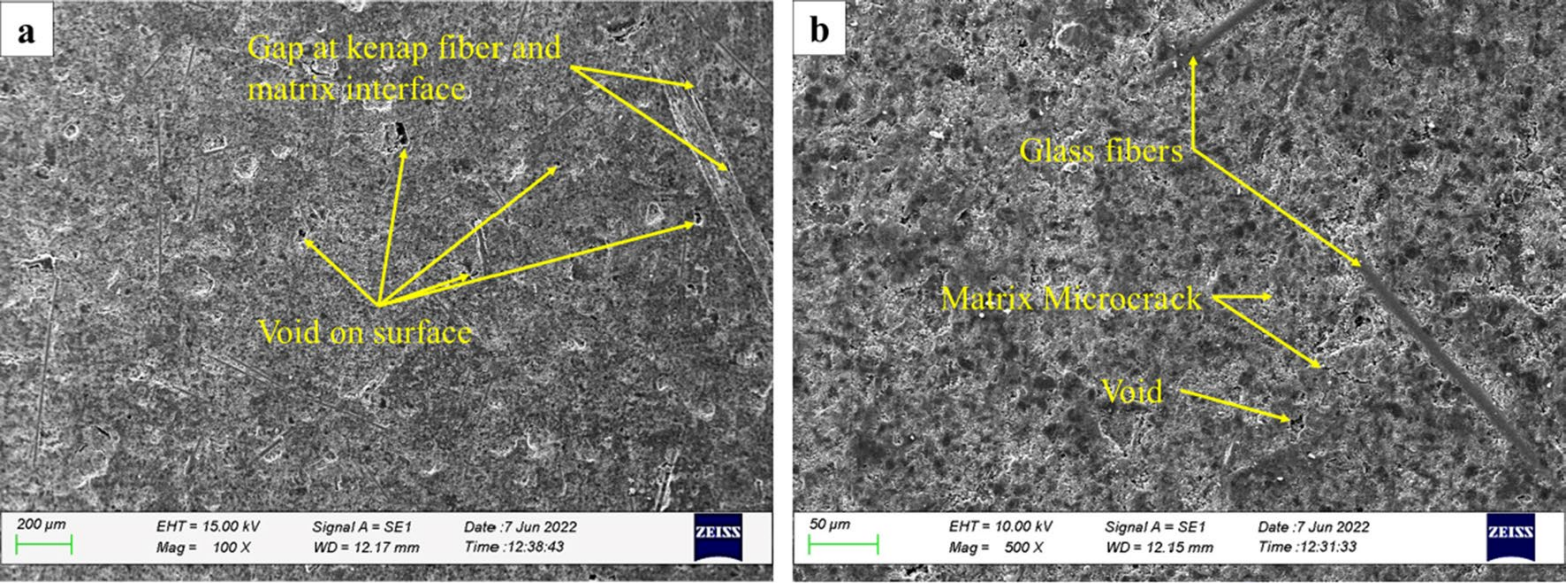
Scanning electron micrographs of the control composites pre-immersion: (**a**) MCC0 and (**b**) MCC12.

A scanning electron microscope (SEM) was used to observe the effect of immersion on the surface morphology of the composites. The surfaces of the MCC0 and MCC12 composites were examined pre- and post-immersion to determine the behaviour of the composites. Figures 9a and b depict the surface morphology of the MCC0 and MCC12 composites pre-immersion.

As seen in Fig. 9, both the MCC0 and MCC12 composites contained cavities, micro-cracks, and fibre-matrix interface gaps. Defects on the surface of the composites may be caused by fabrication failure during the manufacturing process. According to Sanjeevi et al.^8^, during immersion, water molecules enter the composite through cracks or voids followed by entry into the fibre. Therefore, the surface defects observed in these composites may have been caused by the transport of water molecules into the composite.

**Figure 10:**
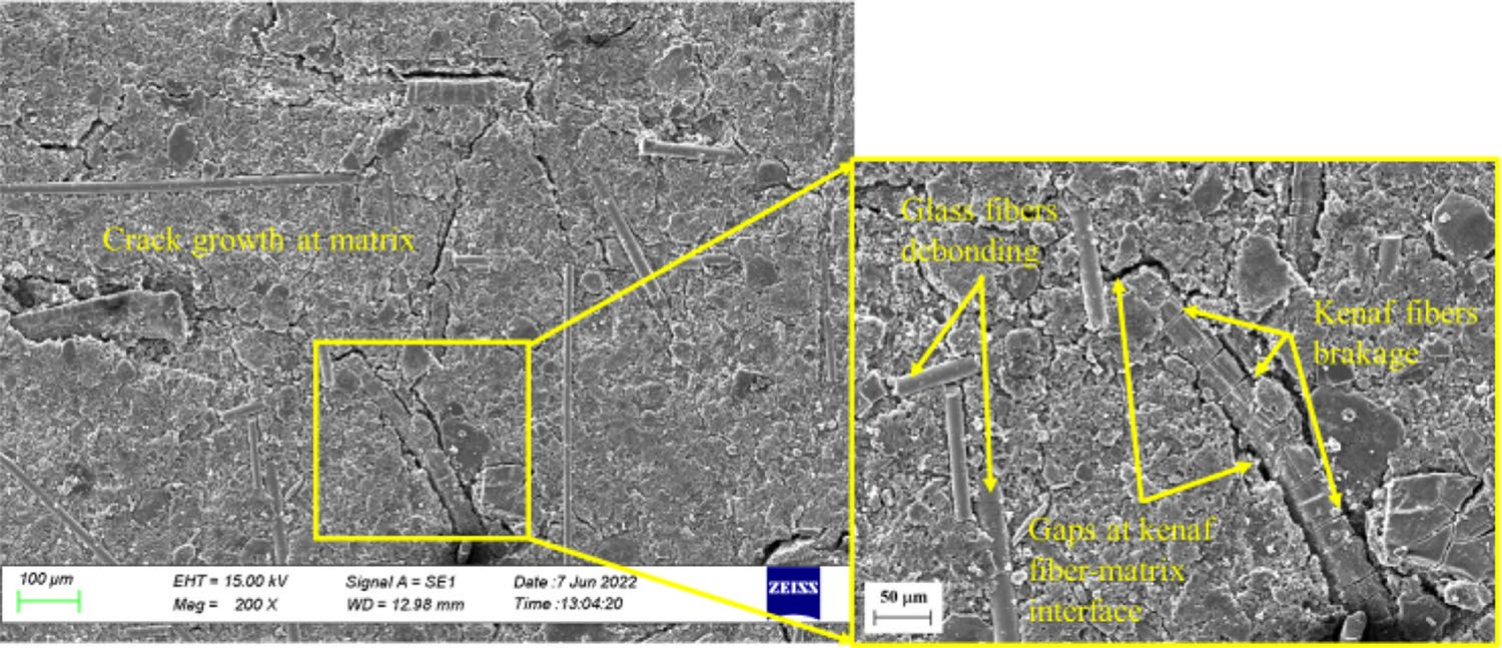
Scanning electron micrographs of the surface of the MCC0 composite post-immersion in distilled water.

Figure 10 depicts the surface defects of the composites post-immersion in distilled water. As seen in Fig. 10, crack growth on the surface of the MCC0 composite post-immersion in distilled water was much higher than pre-immersion (Fig. 9). Furthermore, as glass fibres had not bonded to the resin, along the interface of the kenaf fibres, and the matrix, it indicates the presence of micro-gaps^7^. This phenomenon may be due to water absorption into the composites by constituents that tend to absorb water. The MCC0 composite had the maximum water absorption and the highest *D* (Fig. 6) while the MCC12 composite had the lowest. The increased and faster weight gain may be attributed to the diffusion of water into the material, which degrades the fibre-matrix interface and accelerates moisture-induced interfacial cracking.

Water absorption results in a volumetric expansion that places stress on the matrix, which facilitates crack propagation and the formation of new cracks^47,48^. As seen in Fig. 10, material is lost, most likely in resin, when cracks develop^48^. This breakdown in the composite increases the activity of the water transportation mechanism. As seen in Fig. 10, the glass fibre interface and the matrix de-bond. This debonding decreases the strength of the composite and inhibits the function of the kenaf fibres as a reinforcing binder. When kenaf fibres absorb large quantities of water, transverse fractures form on the surface of the kenaf fibres. Furthermore, the high penetration of water molecules into the kenaf fibre accelerates swelling while prolonged immersion causes the cellulose to degrade^3,28,48^. In this present study, the transverse fractures seen in the kenaf fibres are believed to have occurred due to the degradation of the cellulose because of water molecule penetration into the cellulose during the immersion time.

**Figure 11:**
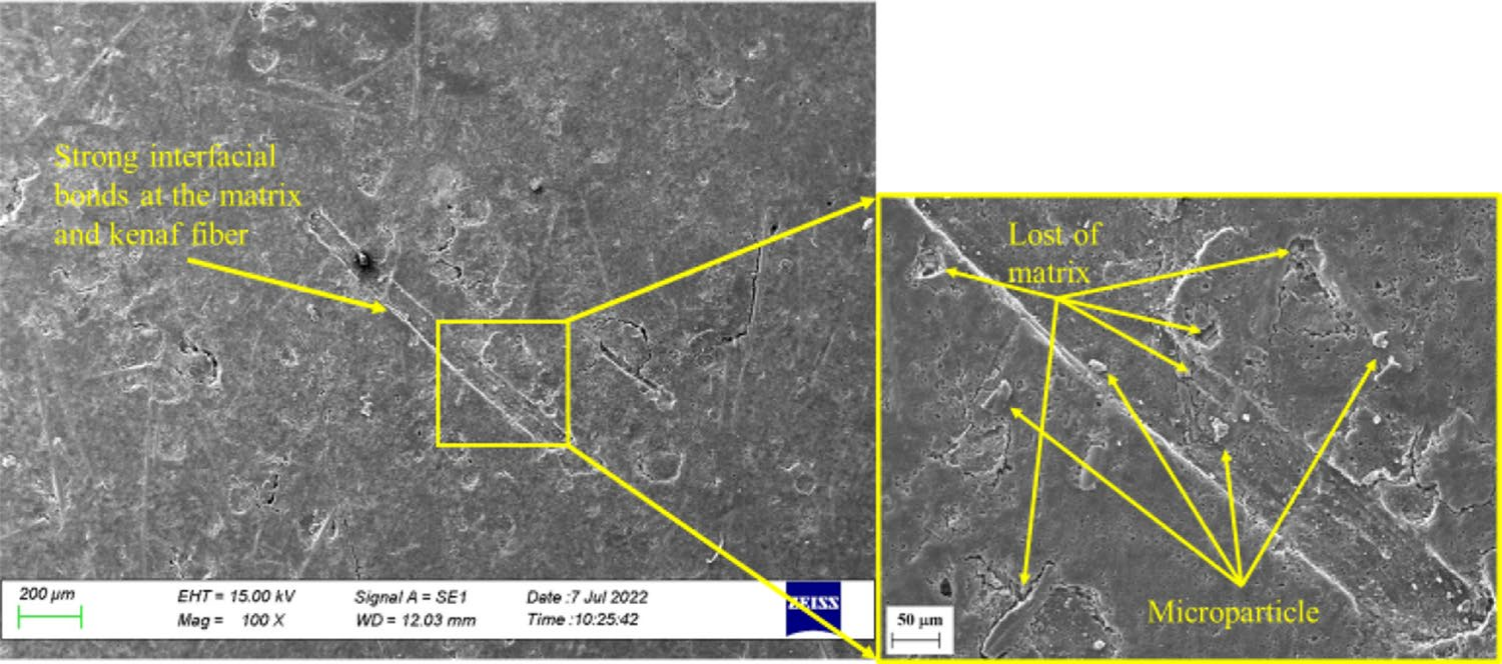
Scanning electron micrographs of the surface of the MCC12 composite post-immersion in distilled water.

As seen in Fig. 11, the bond between the matrix and kenaf fibres on the surface of the MCC12 composite was strong. However, micro-cracks developed on the surface of the matrix post-immersion in distilled water. This was evidenced by the release of microparticles, which indicates polymer degradation on the surface of the material. This also confirmed the findings shown in Fig. 7 and Table 5, where the, respective, curve and percentage of change in composite dimensions (negative *h*_*m*_) indicated a reduction in volume.

**Figure 12:**
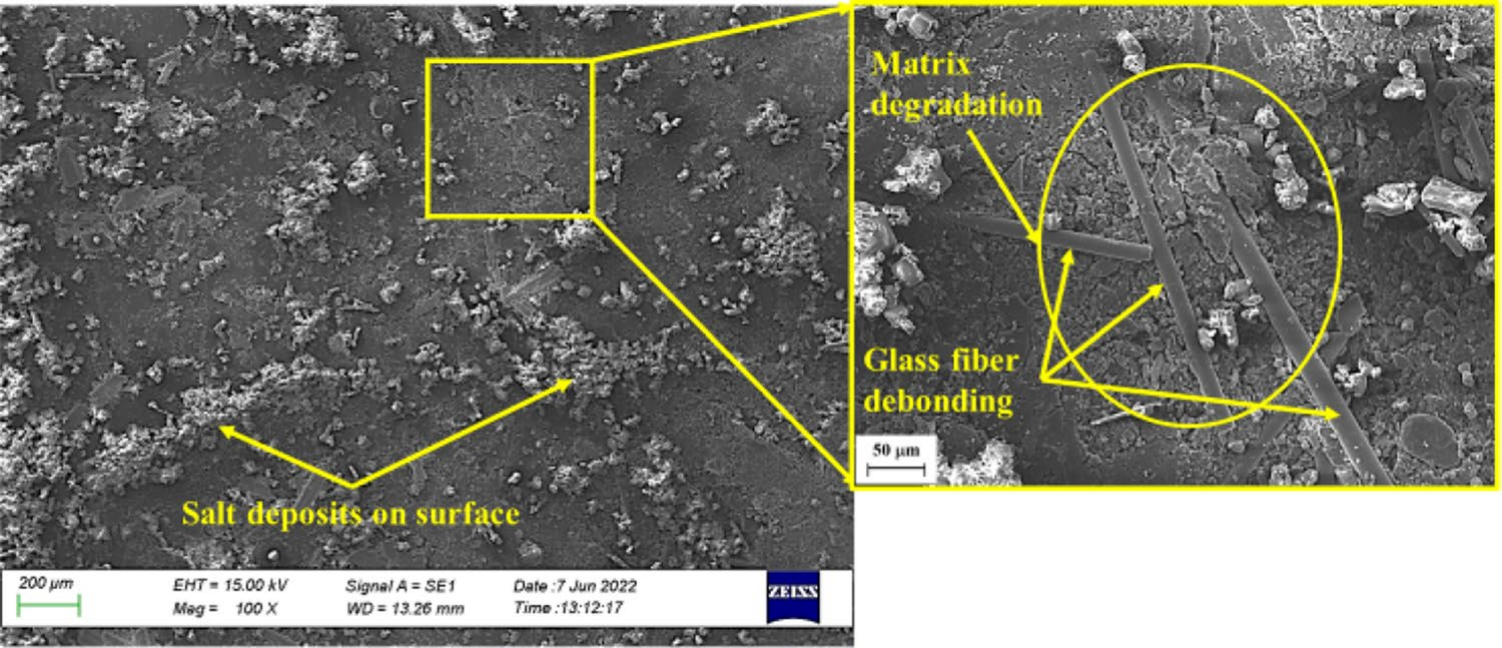
Scanning electron micrographs of the surface of the MCC0 composite post-immersion in seawater.

**Figure 13:**
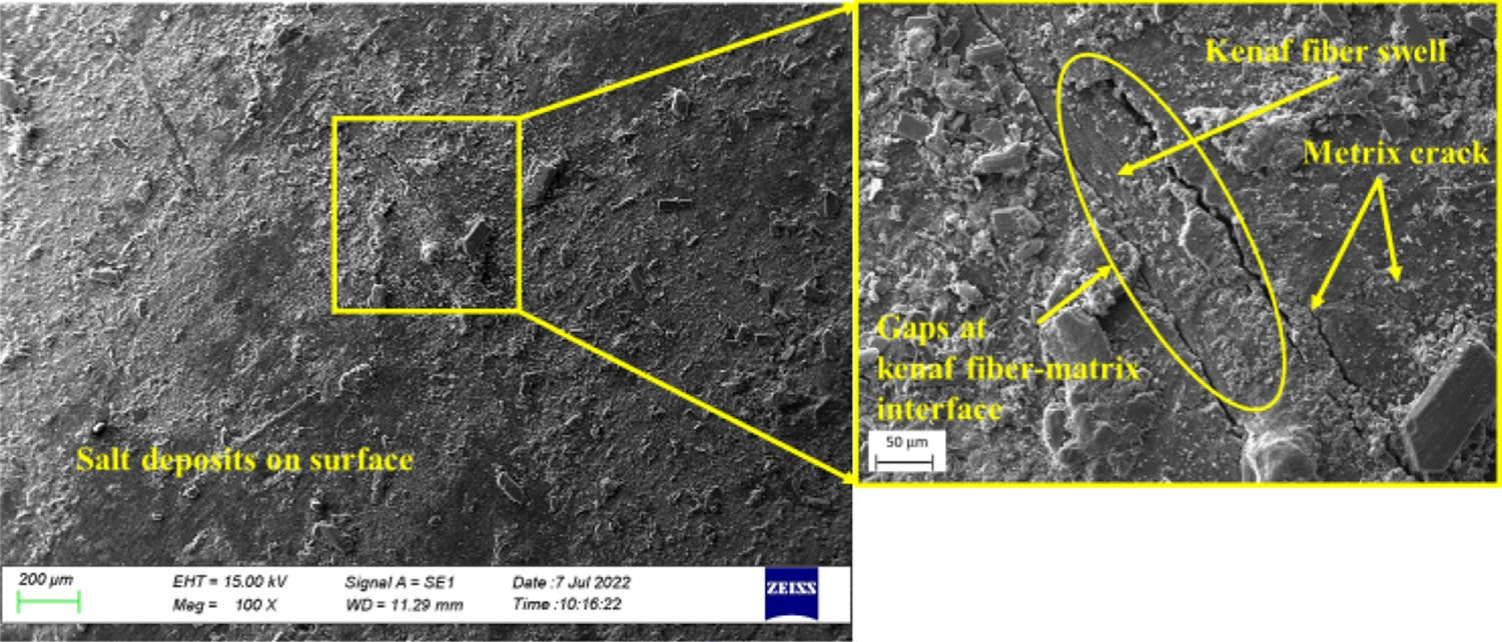
Scanning electron micrographs of the surface of the MCC12 composite post-immersion in seawater.

Polymer degradation occurs when the polymer reacts with the reactant water via the oxidation process. Here, the reaction rate is determined by the 'concentration' of the two reactive pairs^46^. The MCC12 composite had fewer surface defects than the MCC0 composite as it contains a higher vf of MCC, which increases its resistance to oxidation in humid environments^49^, As seen in Fig. 8 and Table 6 the *D* and *M*_*max*_ decreased in the presence of MCC as a composite filler. Meanwhile, as seen in Figs. 12 and 13, a salt solution formed on the surface of the composites post-immersion in seawater.

The formation of salt deposits on the surface are believed to inhibit the diffusion of seawater more than distilled water. Nosbi et al.^3^ similarly reported that the ionic salts found in seawater obstruct the diffusion pathway after long-term exposure, thereby, slowing the absorption process. The findings depicted in Fig. 12 confirm that the diffusion of seawater is less than that of distilled water (Table 6). However, the *M*_*m*_ of seawater was close to that of distilled water as salt deposits accumulated on the surface of the composites. The surface of all degradable polymers erode or lose material upon degradation^50^. As seen in Fig. 12, matrix degradation was indicated by the debonding of the glass fibres, the formation of additional cracks, and surface erosion, which is characterised by increased surface roughness post-immersion (Fig. 9). This is thought to occur as the phenolic resins age faster post-immersion in seawater than in distilled water.

Figure 13 depicts the surface of the MCC12 composite post-immersion in seawater. At 100 × magnification, debonding at the interface of the kenaf fibres and matrix as well as micro-cracks in the surface of the matrix were observed. At 500 × magnification, a gap was observed at the kenaf fibres and matrix interface. This gap may have formed due to the swelling of the kenaf fibres because of the large quantity of water that they absorbed. Meanwhile, the interface of the kenaf fibres and matrix may have de-bonded as the drying process eliminates the moisture content of the kenaf fibres resulting in shrinkage^51^. New micro-cracks formed around the kenaf fibres due to volumetric expansion as the swollen kenaf fibres pressed into the matrix and the bulk constituents of the composite. Fibres swell due to water penetration, leading to cracks in the bulk material and the debonding of the fibre-polymer interface^47^.

The MCC0 and MCC12 composites represent the behaviour of composites with MCC fillers immersed in seawater. The surface erosion and material loss of the MCC12 composite was less than that of the MCC0 composite. This could be due to the lower *D* and *Mm* of the MCC12 composite as it contains more MCC filler (Fig. 8 and Table 6).

**Figure 14:**
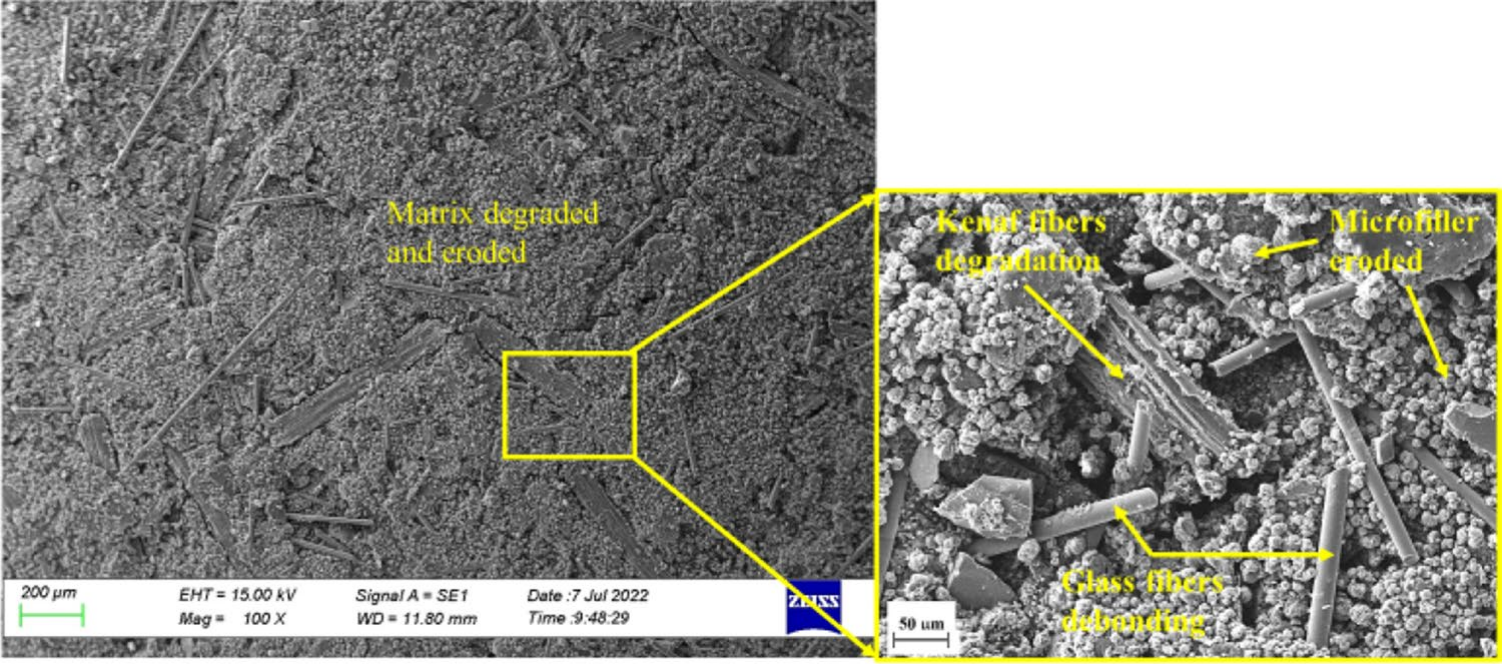
Scanning electron micrographs of the surface of the MCC0 composite post-immersion in the acidic solution.

**Figure 15:**
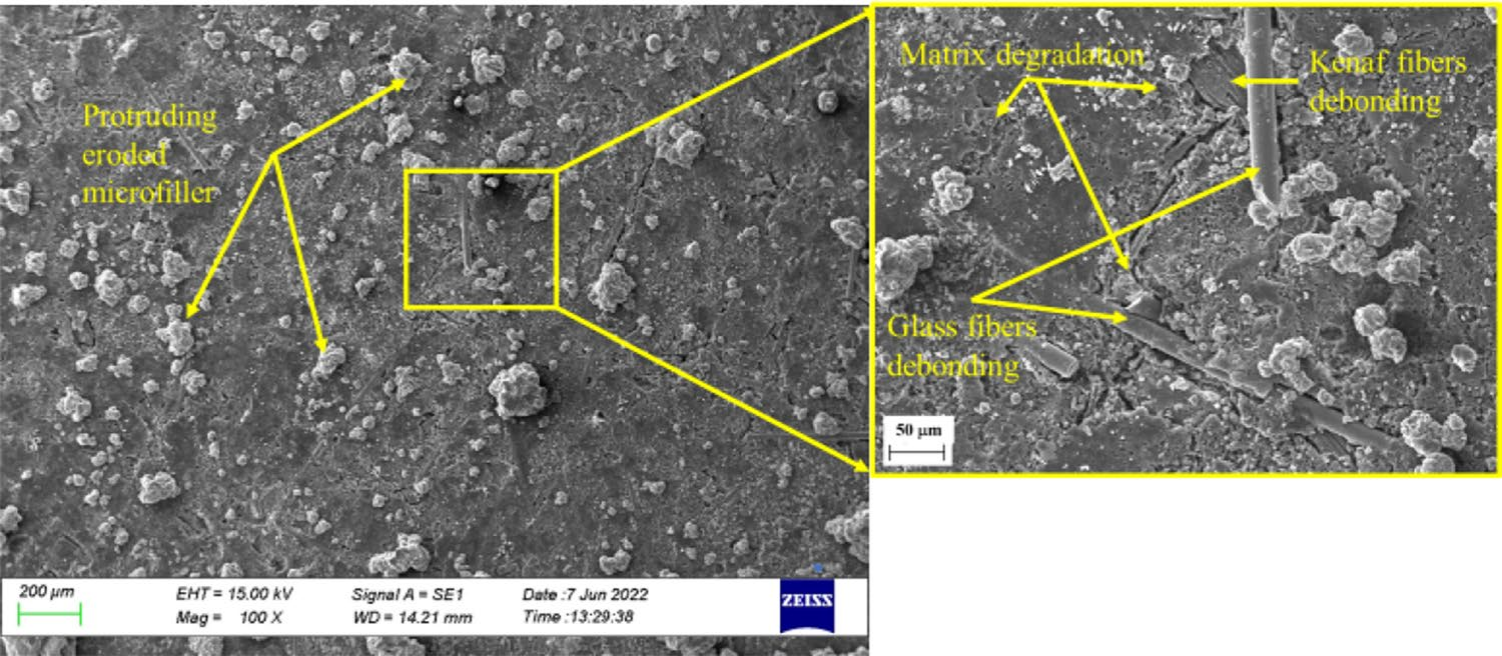
Scanning electron micrographs of the surface of the MCC12 composite post-immersion in the acidic solution.

The SEM micrographs shown in Figs. 14 and 15 indicate the acetic acid immersion changed the surfaces of all the composites. As seen in Fig. 14, micro filler particles had eroded at multiple areas of the surface of the MCC0 composite. Some cavities and micro-cracks had also formed on the surface. Tursiss et al.^52^, similarly, found some micro filler particles protruding from the surface of the composite post-immersion in an acidic solution. This was attributed to the degradation of the polymer matrix. Multiple voids had also formed on the surface, possibly due to the degradation of the resin matrix and loss of surrounding filler particles. Therefore, the changes observed on the surfaces of the MCC0 and MCC12 composites, which consist of a phenolic resin matrix, could be cause by the degradation and erosion of the polymer matrix. These degraded matrix and filler particles are water soluble while the glass fibres are insoluble.

The degradation process is described as a chain cutting process, in which polymer chains are cleaved to form oligomers and monomers. The occurrence of erosion indicates material loss as monomers and oligomers leave the polymer^53^. This passive breaking of the polymer bonds occurs due to hydrolysis and is either caused by water or water as a reactant. Meanwhile, the speed of the reaction depends on the chemistry, solubility, acidity, and oxidation–reduction of each compound^3^. In terms of pH as a degradation medium, neutral pHs have the highest breaking strength of polymer bonds while low and high pHs cause rapid degradation to occur^54^. The matrix degradation observed on the surfaces seen in Figs. 14 and 15 may be attributed to the acidic solution (pH 3) acting as a catalyst that accelerates the reaction of the degradation process of the binding phenolic resin. As seen in Fig. 15, far fewer micro filler particles appeared on the surface of the MCC12 composite than the MCC0 composite. This may have occurred as the MCC filler-reinforcement increases the chain bond strength of the polymer and reduces the likelihood of degradation occurring.

### Flexural properties of the control and immersed MCC composites

**Figure 16:**
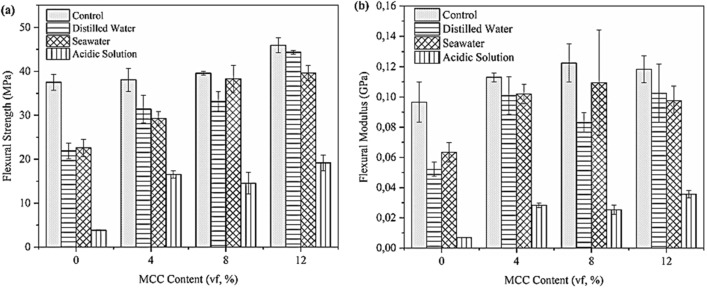
Plot curve of the (**a**) flexural strength and (**b**) flexural modulus of the composites.

Figure 16 depicts the flexural strength and modulus of composites containing no to various vfs of MCC post-immersion in distilled water, seawater, and the acidic solution at 60 days.

As seen in Fig. 16a, the addition of MCC increased the flexural strength of the control and immersed MCC composites. This indicates a strong interaction between the MCC and the phenolic resin matrix via van der Waals forces that facilitate the transfer of stress^55^. In the event of composite failure due to external loads, the failure begins at the pores in the matrix-rich area and continues to form micro-cracks. These cracks then propagate, causing the stress to be transferred to the fibres of the reinforcement matrix, until the composite completely fails. The addition of an MCC filler-reinforcement fills the gaps between the phenolic resin matrix and the fibre. This decreases the likelihood of crack initiation and propagation in the matrix by increasing the amount of energy that the composite can absorb before it fails.

Figure 16b presents the flexural modulus of glass-kenaf fibre reinforced phenolic resin composites containing various vfs of MCC post-immersion in three different mediums. The flexural modulus increased as the MCC vf increased to 8%. However, increasing the MCC vf to 12% decreased the flexural modulus of the composites. This may be due to the solid hydrophilic nature of MCC, which tends easily fuse together via hydrogen bonding, thereby causing agglomeration to occur. This agglomeration inhibits the dispersion and distribution of MCC particles in the matrix^56^. These agglomerated MCC particles then use molecular chains to co-crystallise with the matrix and form stable network structures. This rigid network limits the movement of the molecular chains of the matrix as it lowers the maximum modulus of elasticity and flexibility.

### SEM–EDS elements mapping of flexural fracture

**Figure 17:**
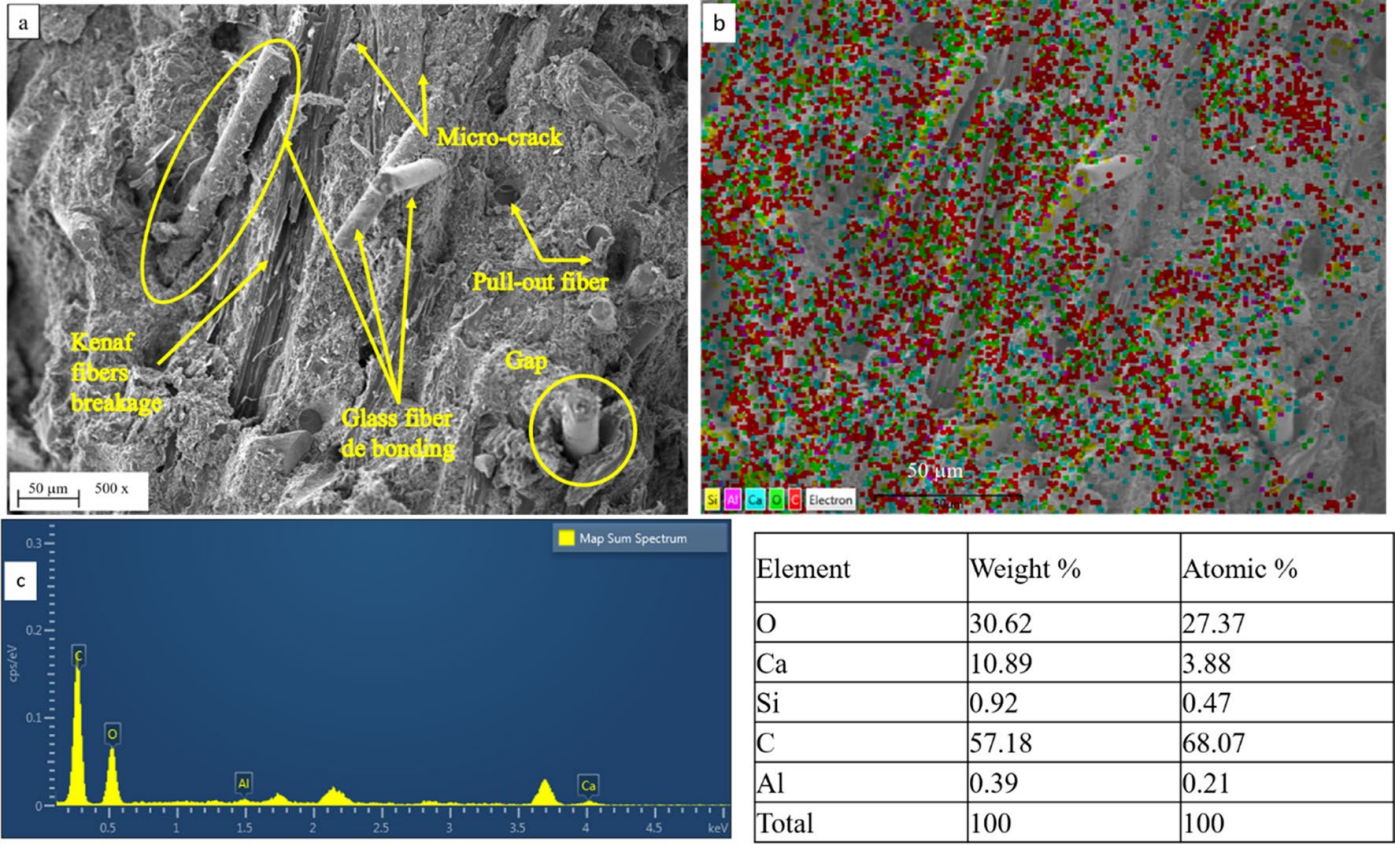
SEM–EDS elemental mapping of flexural fractures in the MCC0 composite, where (**a**) SEM micrographs of flexural fracture on the surface, (**b**) EDS elemental map of composition, and (**c**) EDS spectrum and right table of the atomic and weight percentages of various elements.

**Figure 18:**
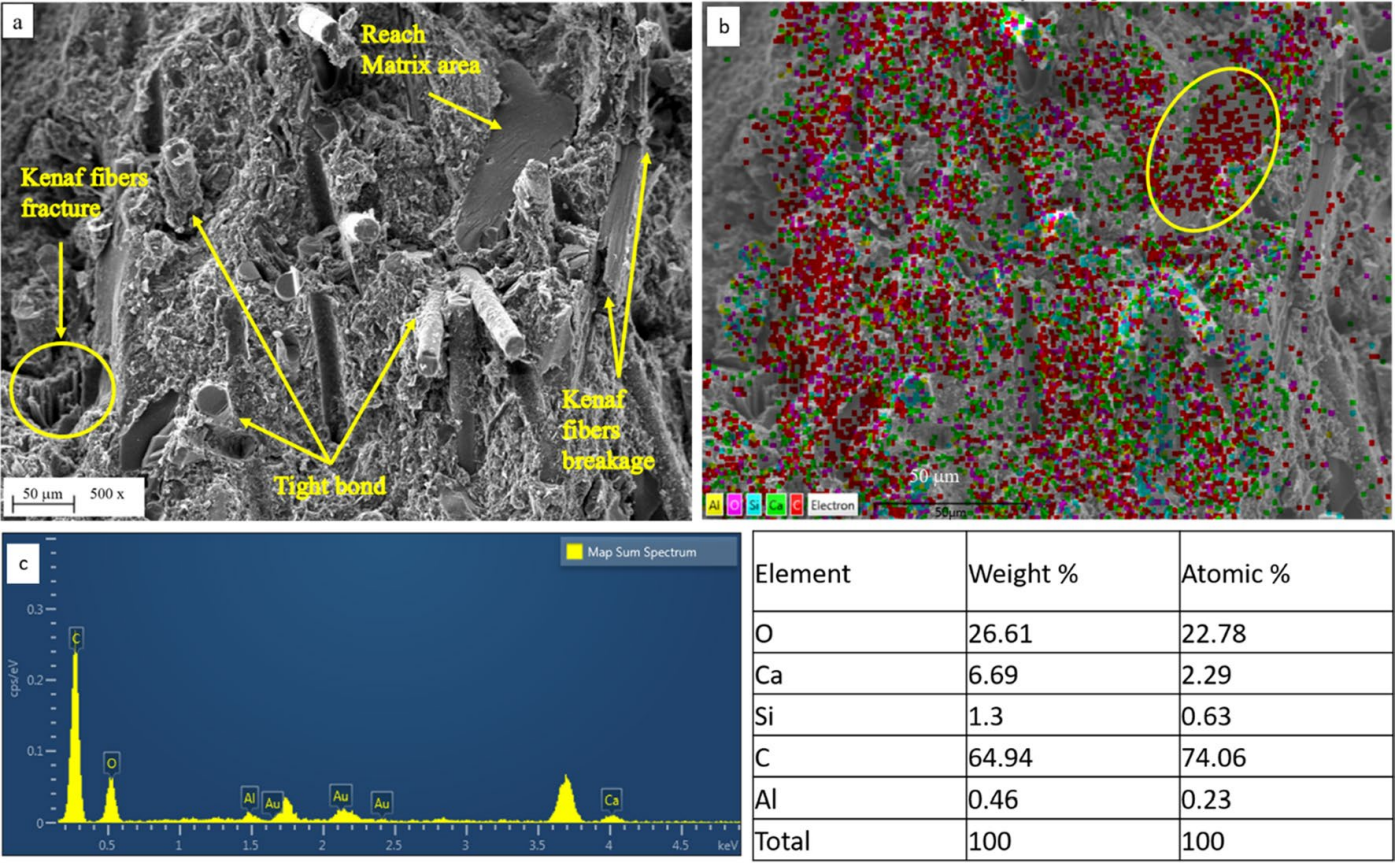
SEM–EDS elemental mapping of flexural fractures in the MCC12 composite, where (**a**) SEM micrographs of flexural fracture on the surface, (**b**) EDS elemental map of composition, and (**c**) EDS spectrum and right table of the atomic and weight percentages of various elements.

Figures 17 and 18 depict the SEM–EDS elemental mapping of flexural fractures in the MCC0 and MCC 12 composites.

As seen in Fig. 17, flexural failure occurred in the MCC0 composite due to the formation of micro-cracks in the resin, defects in the interface, and traces of pull-out in the fibres. The micro cracks observed at the kenaf fibre interface, debonding, and gaps in the glass-kenaf fibre interface indicate weak fibre-matrix interfacial bonding. Furthermore, the presence of pull-out and de-bonded fibres indicate that the fibres had some strength but poor matrix bonding. This caused the composite to poorly resist flexural loads.

As seen in Fig. 18a, flexural failure occurred in the MCC12 composite due to the presence of fractured kenaf fibres, tight interfacial bonding of the glass fibres, and fractures in matrix-rich regions. The presence of fractures in the kenaf fibres indicate a strong bond between the fibre-matrix interface. Meanwhile, the presence of broken kenaf fibres indicate the strength of the kenaf fibre structure to withstand bending loads prior to damage. As the surface of the glass fibres had detached from the resin, it indicates substantial resin adherence to the surface of the glass fibres. Therefore, the glass fibre matrix indicates the strong resistance at the glass fibre-matrix interface and its ability to withstand significant flexural loads prior to failure.

The elements of the MCC0 and MCC12 composites were mapped and compared using SEM/EDX. Figures 17b and 18b depict the EDS spectra, elemental mapping, and secondary electron micrographs of the MCC0 and MCC12 composites, respectively. A spot EDS of granules from each composite indicated that they all contained the amounts of oxygen (O), calcium (Ca), silicon (Si), carbon (C), and aluminium (Al) expected from glass-kenaf fibre composites with thermoset matrices. The table on the right of Figs. 17c and 18c show the atomic percentages and weights of the EDS spectrum elements that the MCC0 and MCC12 composites contain. The MCC12 composite contained less calcium carbonate (CaCO_3_) and more C. This was in accordance with the composite fabrication plan, as the CaCO_3_ acts as a compensator for the presence of the MCC composite filler. The spot EDS of the MCC12 composite (Fig. 18b) indicates the agglomeration of C and O. This agglomeration may be due to the matrix-rich area seen in Fig. 18a. More specifically, the matrix-rich area may have formed as the agglomerated MCC particles interacted with the matrix to form strong bonds^21^. As seen in Figs. 15 and 16, good MCC-matrix interaction increases the strength and bending modulus of the composites.

## Conclusion

It is essential to increase the durability of glass-kenaf fibre reinforced phenolic resin composites submerged in water for its long-term application. This present study discovered that composites that had been immersed in distilled water, seawater, and an acidic solution for 60 days exhibit different behaviours. Composites that had been immersed in distilled water and seawater absorbed water via Fickian diffusion. Composites immersed in distilled water absorbed the most water. When kenaf fibres absorb large amounts of water, it causes them to swell. This, in turn, causes the surface of the kenaf fibres to break, cracks to propagate, and new cracks to form in the matrix. Salt deposits were observed on the surface of composites submerged in seawater. This caused the resin to erode, crack to form, and the loss of some of the resin. Meanwhile, composites immersed in the acidic solution lost mass, which only increased as the immersion duration increased. Micro filler particles were also found to have eroded from the surface. Furthermore, micro-cracks and several cavities appeared on the surface and inside the composite.

As exposure to moisture degraded the fibre-matrix interfaces of the composites, it significantly decreased their flexural properties and flexural modulus. However, the addition of MCC significantly altered the water absorption behaviour of the composites. The addition of MCC, as a filler in thermoset composites reinforced with glass-kenaf fibres, increased interfacial interactions between the matrix and the constituents. Meanwhile, the use of 4%, 8%, and 12% vfs of MCC as a filler-reinforcement decreased the water absorption and mass loss of the composites and increased their flexural strength and flexural modulus.

## Data Availability

The data presented in this study are available on request from the corresponding author.

## Abbreviations

MCC: Micro-crystalline cellulose
KGFRP: Kenaf/glass fiber reinforced polymer
MCC0: KGFRP composites with 0% MCC volume fraction
MCC4: KGFRP composites with 4% MCC volume fraction
MCC8: KGFRP composites with 8% MCC volume fraction
MCC12: KGFRP composites with 12% MCC volume fraction

## Symbols

Sp: Actual mass of the composite (gram)
A: Mass of the test sample without ballast in air (gram)
B: Mass of the test sample and ballast submerged (gram)
W: Actual mass of the ballast submerged (gram)
ρ_a_: Actual density of the composite (g/cm^3)^
*Vv*: Void volume fraction (%)
ρ_1_, ρ_2_,… ρ_10_: Density component (g/cm^3^)
V_1_,V_2_,…V_10_: Volume fraction component (%)
pH: Power of Hydrogen
Mm: The water absorption concentration (%)
Mt: Water absorption at time (%)
M0: Initial mass (gram)
hm: Dimension change (%)
ht: Final thickness (Mm)
h_0_: Initial thickness (Mm)
Mmax: Water absorption at the saturation point (%)
ML: Loss of mass (%)
t: Immersion time (second)
h: Thickness of the sample (Mm)
D: The diffusion coefficient (m.s^-1/2)^
n: Kinetic parameters of the slope
k: Kinetic parameters of the interseception
ASTM: American Standard Testing and Material
SEM: Scanning Electron Microscope
EDX: Energy-dispersive X-ray spectroscopy
EDS: Energy dispersive spectroscopy

## References

[1] Afzaluddin A, Jawaid M, Sapuan M (2018). Physical and mechanical properties of sugar palm / glass fiber reinforced thermoplastic polyurethane hybrid composites. J. Mater. Sci. Res Technol..

[2] Khalid MY, Al Rashid A, Arif ZU, Akram N, Arshad H, Márquez FPG (2021). Characterization of failure strain in fiber reinforced composites: Under on-axis and off-axis loading. Crystals.

[3] Nosbi N, Akil HM, Mohd Ishak ZA, Abu Bakar A (2009). Water absorption behavior of pultruded kenaf fiber reinforced unsaturated polyester composites and its effects on mechanical properties. ICCM Int. Conf. Compos. Mater..

[4] Manjunath RN, Khatkar V, Behera BK (2020). Investigation on Seawater Ageing of PET-Epoxy Composites: An Ecological and Sustainable Approach for Marine Applications. J. Polym. Environ..

[5] Abdurohman K, Adhitya M (2021). Effect of seawater immersion on mechanical properties of glass/vinylester composites for marine application. AIP Conf. Proc..

[6] Chakraverty AP, Mohanty UK, Mishra SC, Satapathy A (2015). Sea water ageing of GFRP composites and the dissolved salts. IOP Conf. Ser. Mater. Sci. Eng..

[7] Asfar MF, Ariawan D, Triyono J (2019). Effect of immersion on the impact properties of Zalacca midrib fiber/rHDPE by compression molding. AIP Conf. Proc..

[8] Sanjeevi S (2021). Effects of water absorption on the mechanical properties of hybrid natural fibre/phenol formaldehyde composites. Sci. Rep..

[9] Rudawska A (2020). The impact of the acidic environment on the mechanical properties of epoxy compounds in different conditions. Polymers (Basel).

[10] Münchow EA, Ferreira ACA, Machado RMM, Ramos S, Rodrigues-junior SA, Zanchi CH (2014). E f f e c t o f Ac i d i c S o l u t i o n s o n the surface degradation of a micro-hybrid composite resin. Braz. Dent. J..

[11] Lao LL, Peppas NA, Boey FYC, Venkatraman SS (2011). Modeling of drug release from bulk-degrading polymers. Int. J. Pharm..

[12] Alshammari BA, Saba N, Alotaibi MD, Alotibi MF, Jawaid M, Alothman OY (2019). Evaluation of mechanical, physical, and morphological properties of epoxy composites reinforced with different date palm fillers. Materials (Basel).

[13] Mallick PK (2017). Particulate filled and short fiber reinforced polymer composites, 2(June), 2015. Elsevier Ltd..

[14] Rehman MM, Zeeshan M, Shaker K, Nawab Y (2019). Effect of micro-crystalline cellulose particles on mechanical properties of alkaline treated jute fabric reinforced green epoxy composite. Cellulose.

[15] Nurazzi NM (2021). A review on mechanical performance of hybrid natural fiber polymer composites for structural applications. Polymers (Basel).

[16] Treimank A, Laka M, Chernyavskaya S, Erdmann JGJ, Ziegler L, Birska I (2016). Microcrystalline cellulose fillers for use in hybrid composites with polyethylene and lignin. Cellul. Chem. Technol..

[17] Sakuri S, Surojo E, Ariawan D, Prabowo AR (2020). Experimental investigation on mechanical characteristics of composite reinforced cantala fiber (CF) subjected to microcrystalline cellulose and fumigation treatments. Compos. Commun..

[18] Kiziltas A, Gardner DJ, Han Y, Yang HS (2014). Mechanical properties of microcrystalline cellulose (MCC) filled engineering thermoplastic composites. J. Polym. Environ..

[19] Umar M, Mohammed SSD (2011). Chemical modification of microcrystalline cellulose: Improvement of barrier surface properties to enhance surface interactions with some synthetic polymers for biodegradable packaging material processing and applications in textile. Food Pharmaceutica. Pelagia Res. Libr..

[20] Cataldi A, Dorigato A, Deflorian F, Pegoretti A (2014). Effect of the water sorption on the mechanical response of microcrystalline cellulose-based composites for art protection and restoration. J. Appl. Polym. Sci..

[21] Ramires EC, Megiatto JD, Dufresne A, Frollini E (2020). Cellulose nanocrystals versus microcrystalline cellulose as reinforcement of lignopolyurethane matrix. Fibers.

[22] Jamasri V, Malau M, Ilman N, Surojo E (2014). Effect of ingredients on flexural strength of friction composite. Appl. Mech. Mater..

[23] Sukanto H, Raharjo WW, Ariawan D, Triyono J, Kaavesina M (2021). Epoxy resins thermosetting for mechanical engineering. Open Eng..

[24] Hafiz NLM (2020). Curing and thermal properties of co-polymerized tannin phenol-formaldehyde resin for bonding wood veneers. J. Mater. Res. Technol..

[25] B. C. Duncan and W. R. Broughton, (2007) Measurement good practice guide no 102 absorption and diffusion of moisture in polymeric materials. Meas. Good Pract. Guid. no. 102

[26] ASTM D570, (2014) Standard test method for water absorption of plastics. ASTM Stand. vol. 98, no. Reapproved 2010, pp. 25–28, 2014, doi: 10.1520/D0570-98R10E01.2.

[27] Hosseinihashemi SK, Arwinfar F, Najafi A, Nemli G, Ayrilmis N (2016). Long-term water absorption behavior of thermoplastic composites produced with thermally treated wood. Meas. J. Int. Meas. Confed..

[28] Balogun OP, Adediran AA, Omotoyinbo JA, Alaneme KK, Oladele IO (2020). Evaluation of water diffusion mechanism on mechanical properties of polypropylene composites. Int. J. Polym. Sci..

[29] Akil HM, Santulli C, Sarasini F, Tirillò J, Valente T (2014). Environmental effects on the mechanical behaviour of pultruded jute/glass fibre-reinforced polyester hybrid composites. Compos. Sci. Technol..

[30] HestiawanKusmono H, Jamasri, (2020). The water absorption, mechanical and thermal properties of chemically treated woven fan palm reinforced polyester composites. J. Mater. Res. Technol..

[31] Nawangsari P, Jamasri H, Rochardjo SB (2019). Effect of phenolic resin on density, porosity, hardness, thermal stability, and friction performance as a binder in non-asbestos organic brake pad. IOP Conf. Ser. Mater. Sci. Eng..

[32] Li Y, Li Q, Ma H (2015). The voids formation mechanisms and their effects on the mechanical properties of flax fiber reinforced epoxy composites. Compos. Part A Appl. Sci. Manuf..

[33] Jena H, Panigrahi A (2021). The effect of clam shell powder on kinetics of water absorption of jute epoxy composite. World J. Eng..

[34] Costa ML, Rezende MC, de Almeida SFM (2006). Effect of void content on the moisture absorption in polymeric composites. Polym. Plast. Technol. Eng..

[35] Iordanskii AL, Zaikov GE, Berlin AA (2015). Diffusion kinetics of hydrolysis of biodegradable polymers. Weight loss and control of the release of low molecular weight substances. Polym. Sci.Ser. D.

[36] Thoorens G, Krier F, Leclercq B, Carlin B, Evrard B (2014). Microcrystalline cellulose, a direct compression binder in a quality by design environment-A review. Int. J. Pharm..

[37] Larsson M, Johnsson A, Gårdebjer S, Bordes R, Larsson A (2017). Swelling and mass transport properties of nanocellulose-HPMC composite films. Mater. Des..

[38] dos Santos FA, Iulianelli GCV, Tavares MIB (2017). Effect of microcrystalline and nanocrystals cellulose fillers in materials based on PLA matrix. Polym. Test..

[39] Deroiné M (2014). Accelerated ageing of polylactide in aqueous environments: Comparative study between distilled water and seawater. Polym. Degrad. Stab..

[40] Valinoti AC, Neves BG, Da Silva EM, Maia LC (2008). Surface degradation of composite resins by acidic medicines and pH-cycling. J. Appl. Oral Sci..

[41] Taib RM, Ariawan D, Ishak ZAM (2014). Effects of alkali treatment on the properties of kenaf fiber-unsaturated polyester composites prepared by resin transfer molding. Mol. Cryst. Liq. Cryst..

[42] deVNaylor T (1989). Permeation properties. Compr. Polym. Sci. Suppl.

[43] Wong EH, Chan KC, Lim TB, Lam TF (1999). Non-Fickian moisture properties characterisation and diffusion modeling for electronic packages. Proc. - Electron. Components Technol. Conf..

[44] Seera SDK, Kundu D, Banerjee T (2020). Physical and chemical crosslinked microcrystalline cellulose-polyvinyl alcohol hydrogel: Freeze–thaw mediated synthesis, characterization and in vitro delivery of 5-fluorouracil. Cellulose.

[45] Zuhudi NZM, Zulkifli AF, Zulkifli M, Yahaya ANA, Nur NM, Aris KDM (2021). Void and moisture content of fiber reinforced composites. J. Adv. Res. Fluid Mech. Therm. Sci..

[46] Göpferich A (1997). Polymer bulk erosion. Macromolecules.

[47] Otaluka EP, Arnold C, Alston S (2015). The long term effects of water absorption, desorption abd reabsorption in carbon-fibre. 10th Int. Conf. Compos. Sci. Technol..

[48] Dhakal HN, Zhang ZY, Richardson MOW (2007). Effect of water absorption on the mechanical properties of hemp fibre reinforced unsaturated polyester composites. Compos. Sci. Technol..

[49] Lv J, Chen ZL (2021). Analysis the performance of hydrophilic and corrosion resistant coatings on the distillation desalination tube in high temperature seawater. IOP Conf. Ser. Earth Environ. Sci..

[50] Göpferich A (1996). Mechanisms of polymer degradation and erosion1. Biomater. Silver Jubil. Compend..

[51] Gautier L, Mortaigne B, Bellenger V (1999). Interface damage study of hydrothermally aged glass-fibre-reinforced polyester composites. Compos. Sci. Technol..

[52] Turssi CP, Hara AT, Serra MC, Rodrigues AL (2002). Effect of storage media upon the surface micromorphology of resin-based restorative materials. J. Oral Rehabil..

[53] Blackwell CJ, Haernvall K, Guebitz GM, Groombridge M, Gonzales D, Khosravi E (2018). Enzymatic degradation of star poly(ε-caprolactone) with different central units. Polymers (Basel).

[54] Vaid R, Yildirim E, Pasquinelli MA, King MW (2021). Hydrolytic degradation of polylactic acid fibers as a function of ph and exposure time. Molecules.

[55] Chen Q, Shi Y, Chen G, Cai M (2020). Enhanced mechanical and hydrophobic properties of composite cassava starch films with stearic acid modified MCC (microcrystalline cellulose)/NCC (nanocellulose) as strength agent. Int. J. Biol. Macromol..

[56] Mohan Bhasney S, Kumar A, Katiyar V (2019). Microcrystalline cellulose, polylactic acid and polypropylene biocomposites and its morphological, mechanical, thermal and rheological properties. Compos. Part B Eng..

